# ﻿Island hoppers: Integrative taxonomic revision of *Hogna* wolf spiders (Araneae, Lycosidae) endemic to the Madeira islands with description of a new species

**DOI:** 10.3897/zookeys.1086.68015

**Published:** 2022-02-16

**Authors:** Luís C. Crespo, Isamberto Silva, Alba Enguídanos, Pedro Cardoso, Miquel Arnedo

**Affiliations:** 1 Department of Evolutionary Biology, Ecology and Environmental Sciences (Arthropods), Biodiversity Research Institute (IRBio), Universitat de Barcelona, Av. Diagonal 643, 08028 Barcelona, Spain Universitat de Barcelona Barcelona Spain; 2 Laboratory for Integrative Biodiversity Research (LIBRe), Finnish Museum of Natural History (LUOMUS), University of Helsinki, P.O. Box 17, 00014 Helsinki, Finland University of Helsinki Helsinki Finland; 3 Instituto das Florestas e Conservação da Natureza IP-RAM, Jardim Botânico da Madeira, Caminho do Meio, Bom Sucesso, 9064-512, Funchal, Portugal Instituto das Florestas e Conservação da Natureza IP-RAM, Jardim Botânico da Madeira Funchal Portugal

**Keywords:** Endangered species, island radiation, Lycosinae, Macaronesia, morphological polymorphism, species delimitation

## Abstract

Because of their ability for aerial dispersal using silk and preference for open habitats, many wolf spiders are formidable colonisers. Pioneering arachnologists were already aware of the large and colourful wolf spiders in the Madeira archipelago, currently included in the genus *Hogna* Simon, 1885. The origins were investigated and species boundaries of Madeiran *Hogna* examined by integrating target-gene and morphological information. A multi-locus phylogenetic analysis of a thorough sampling across wolf-spider diversity suggested a single origin of Madeiran endemics, albeit with low support. Divergence time estimation traced back their origin to the late Miocene, a time of major global cooling that drove the expansion of grasslands and the associated fauna. Morphological examination of types and newly collected material revealed a new species, hereby described as *H.isambertoi* Crespo, **sp. nov.** Additionally, *H.blackwalli* is revalidated and three new synonymies are proposed, namely *H.biscoitoi* Wunderlich, 1992, junior synonym of *H.insularum* Kulczynski, 1899, *H.schmitzi* Wunderlich, 1992, junior synonym of *H.maderiana* (Walckenaer, 1837), and *Arctosamaderana* Roewer, 1960 junior synonym of *H.ferox* (Lucas, 1838). Species delimitation analyses of mitochondrial and nuclear markers provided additional support for morphological delineations. The species pair *H.insularum* and *H.maderiana*, however, constituted an exception: the lack of exclusive haplotypes in the examined markers, along with the discovery of intermediate forms, pointed to hybridisation between these two species as reported in other congeneric species on islands. Finally, the conservation status of the species is discussed and candidates for immediate conservation efforts are identified.

## ﻿Introduction

Most wolf spiders (Lycosidae) are ground-dwelling cursorial hunters, with only a small portion of its species displaying sheet-web building behaviour. They are one of the most abundant and ubiquitous spiders in open terrestrial habitats, such as grass- and shrublands. It has been suggested that lycosids underwent major global diversification concomitantly with grassland expansion during the Miocene (Jocqué and Alderweireldt 2005; Piacentini and Ramírez 2019). Some groups of wolf spiders frequently use ballooning, a form of passive airborne transport mediated by silk (Bell et al. 2005). The ability for long-distance dispersal combined with their preference for open and disturbed habitats for many species, makes them formidable colonisers of oceanic islands, including the world’s most remote island chain, the Hawaiian Archipelago (Suman 1964). The genus *Hogna* Sundevall, 1833 includes medium- to large-sized spiders said to have a worldwide distribution, although this fact is probably derived from a lack of any recent thorough systematic studies. Despite its size, the genus has managed to colonise and diversify on many oceanic islands, including the Galápagos (Baert et al. 2008) in the Pacific Ocean and Saint Helena, in the south Atlantic (Tongiorgi 1977). Similarly, the Madeira archipelago also harbours several endemic species of *Hogna*. Among spiders, *Hogna* (7 species) is second only to the genus *Dysdera* Latreille, 1804 (11 species) in numbers of endemic species present in the Madeira archipelago (Crespo et al. 2021), and some of its species rank among the most emblematic organisms of the islands.

**Figure 1.**
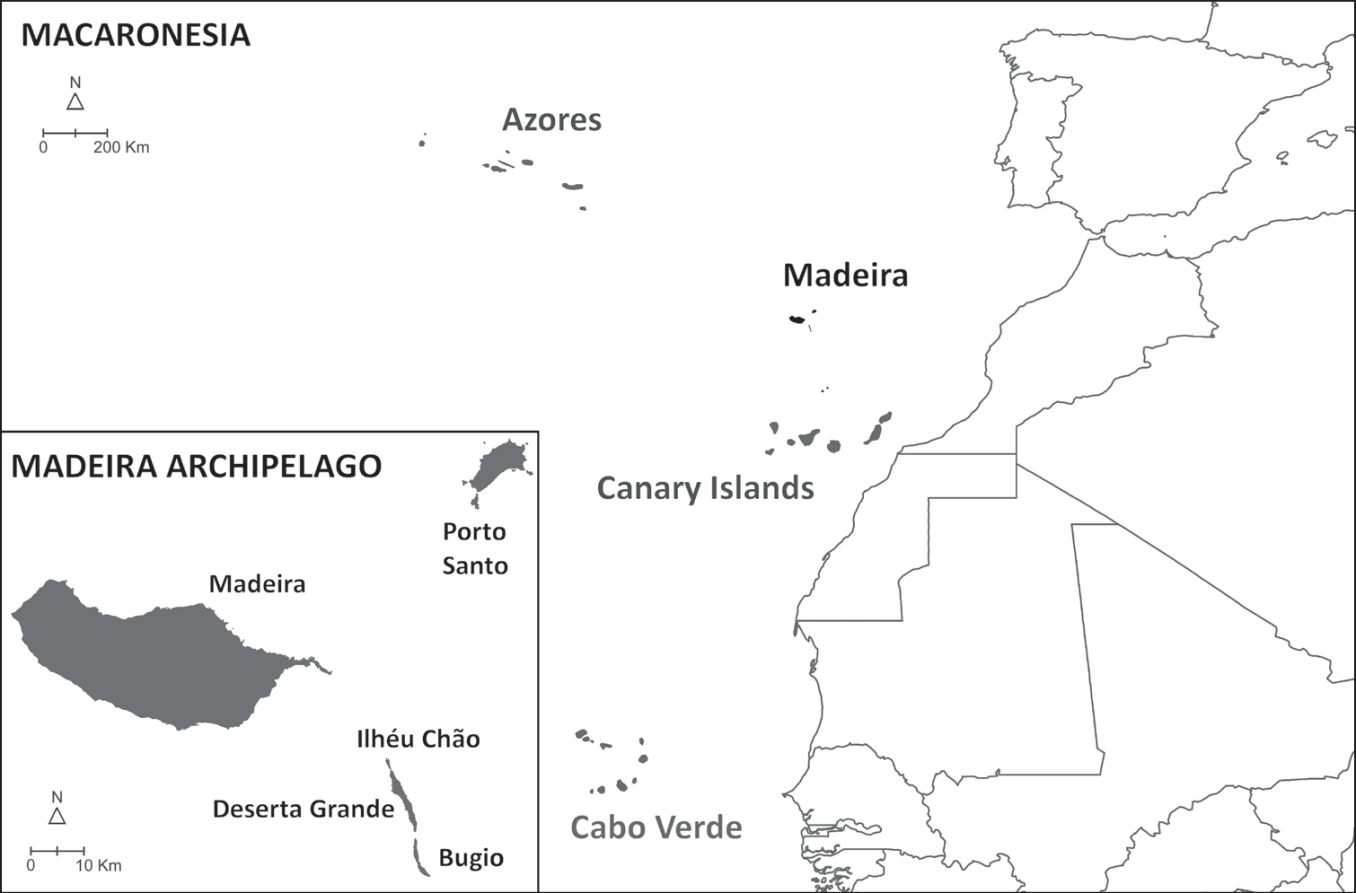
Map of the Macaronesia and the Madeira archipelago (adapted from Borges et al. 2008, with authors’ permission).

Madeira is situated in the North Atlantic Ocean, approximately 500 km north of the Canary Islands, 900 km west from Morocco, and 1000 km southwest from the Iberian Peninsula (Fig. 1). It is composed of a small number of islands and islets aligned in a southwestern direction as a result of their sequential formation from a volcanic hotspot on the oceanic crust. Among the larger islands, Porto Santo is a small and relatively flat island (maximum altitude 516 m at Pico do Facho), surrounded by several islets in a later stage of island ontogeny, its subaerial stage dating back to 14 million years ago (mya). The emergences of the two other larger islands, Madeira and Deserta Grande, date back to 7 and 5 mya, respectively (Geldmacher and Hoernle 2000; Schwarz et al. 2005; Ramalho et al. 2015). Although both islands are in an intermediate stage of the island ontogeny, they show substantially different geomorphology. Madeira is larger with a rugged, steep orography, especially in its northern side, reaching a maximum altitude of 1861m at Pico Ruivo. This stands at a sharp contrast with the aspect of the Deserta Grande, which together with the islets of Ilhéu Chão and Bugio constitute the Desertas islands, with a maximum altitude of only 479 m (Rocha do Barbusano), yet displaying a dramatic topographic relief, also observed in Bugio. The Madeira islands exhibit a wide variety of habitats, ranging from the humid subtropical laurel forest of Madeira to the *Erica* shrublands, high-elevation and coastal grasslands, or rocky scarps across all islands and islets. Madeiran *Hogna* spiders occur throughout all these habitats, mostly on montane or coastal grasslands and rocky scarps, as is common for the family, but also in closed-canopy laurel forest.

Due to their large size, restricted distribution, and striking appearance of some species, either in size or distinctive leg coloration, local *Hogna* spiders were known to naturalists since the early 19^th^ century. The largest and most colourful species were the first to be described, namely *H.maderiana* (Walckenaer, 1837) and *H.ingens* (Blackwall, 1857). By the end of the 19^th^ century, two smaller species, *H.heeri* (Thorell, 1875) and *H.insularum* (Kulczynski, 1899), were added to the checklist. The report of new endemic *Hogna* species had to wait for almost a century, until the description of *H.biscoitoi* Wunderlich, 1992, *H.schmitzi* Wunderlich, 1992, and *H.nonannulata* Wunderlich, 1995.

Although no other taxonomic work on Madeiran *Hogna* has been published for more than 25 years, a number of taxonomic problems remained to be tackled, including nomenclatural issues and the interpretation of intraspecific variability in the context of intermediate forms (Wunderlich 1992, 1995). In addition, recent studies suggest that species delimitation in wolf spiders may be hampered by either the recent origin of some species (Ivanov et al. 2021) or introgression events among close relatives (De Busschere et al. 2015). On the other hand, the genus *Hogna* is in much need of a thorough revision (Logunov 2020). Brady (2012) has provided a diagnosis based on coloration and eye arrangement, while stating that genitalic morphology, traditionally used by taxonomists to identify species, cannot be used to separate *Hogna* from other Lycosinae genera. Descriptions of the old species are usually vague, poorly illustrated and, in some cases, the type materials have been lost. As a result, the genus has traditionally served as a dumping ground for large lycosids of uncertain placement in the Lycosinae. The lack of a clear circumscription of the genus poses a burden in terms of identifying the putative source of colonisers of the Madeiran species.

Some of the Madeira *Hogna* species are of conservation concern. The Desertas giant wolf spider, *H.ingens*, is listed as “Critically Endangered” on the IUCN Red List of Threatened Species due to its narrow distribution range and the fact that the native vegetation of the small valley it inhabits has been mostly displaced by an invasive grass (Crespo et al. 2014b). Conservation efforts involving an ex-situ breeding program and management control of the grasses are underway (Cardoso et al. 2016).

In the present study, we integrate morphological and natural history information with molecular data to (1) test the monophyly of the Madeiran *Hogna* to resolve the number and timeline of colonisation events, (2) delimitate species boundaries and (3) conduct a taxonomic revision of these iconic endemic species.

## ﻿Materials and methods

### ﻿Field work

The material studied here was made available through collections from expeditions to Madeira, Porto Santo and the Desertas in springs of 2017 and 2018. Additional specimens were provided by occasional collecting by one of us (IS). Sampling was done in a wide variety of habitats, especially in open areas surrounding native vegetation patches, by lifting stones and retrieving *Hogna* specimens manually. Each specimen was placed into a separate cryovial containing 96% molecular grade ethanol and stored in a freezer at -20 °C until further study. Specimens for morphological analyses were later transferred to glass vials containing 75% ethanol. The sampling coordinates, when available, are shown in decimal degrees format.

### ﻿Molecular lab procedures

We extracted DNA from one leg III using commercial kits (Speedtools Tissue DNA Extraction Kit, Biotools; or DNeasy Blood & Tissue Kit, Qiagen) following the tissue protocol suggested by the respective manufacturer. We amplified partial fragments of the mitochondrial cytochrome c oxidase subunit I (COI), i.e., the animal DNA barcode (Hebert et al. 2003), the small ribosomal subunit 12S rRNA (12S), large ribosomal subunit 16S rRNA (16S), the tRNA Leu (L1), the NADH dehydrogenase subunit 1 (nad1), and the nuclear large ribosomal subunit 28S rRNA (28S), the internal transcribed spacer 2 (ITS-2) and the histone 3 (H3) genes. The primers used for amplification and sequencing, as well as the PCR conditions for the loci are listed in Suppl. material 1. The final PCR product was sequenced by Macrogen Inc. (Seoul, South Korea). Sequences were edited and managed in GENEIOUS Prime 2021.0.3 (https://www.geneious.com). Voucher sequence data of samples used in phylogenetic analysis is available and accession numbers are available in Suppl. material 2.

### ﻿Phylogenetic analyses

To test the monophyly and phylogenetic structure of Madeiran *Hogna*, we combined our newly generated sequences with the data matrix of Piacentini and Ramírez (2019) designed to infer phylogenetic relationships for the family Lycosidae using a target gene approach. Additional sequences of *Hogna* species were retrieved from GenBank. We aligned sequence fragments of COI, 12S, 16S-L1, nad1, 28S, and H3 individually per gene using the GENEIOUS plugin of the alignment program MAFFT v. 1.4.0 (Katoh and Standley 2013), using the G-INS-I algorithm with default options. We concatenated all genes in a super matrix for subsequent phylogenetic analyses with the help of the program SEQUENCE MATRIX (Vaidya et al. 2011).

Parsimony analysis of the matrix was conducted with the program TNT v1.5 (Goloboff and Catalano 2016). We first recoded gaps as absence/presence characters using the simple coding method proposed by Simmons and Ochoterena (2000) with the help of the computer program SEQSTATE (Müller 2005). Search strategy for shortest trees combined sectorial searches, tree fusing, drift and ratchet. Tree searches were driven to hit independently 10 times the optimal scoring, followed by Tree Bisection and Reconnection (TBR) branch swapping, saving up to 1000 trees (Soto et al. 2017). We estimated support values by jackknifing frequencies derived from 1000 resampled matrices using 15 random addition sequences, retaining 20 trees per replication, followed by TBR, and TBR collapsing to calculate the consensus. We inferred the best maximum likelihood trees with IQ-TREE v. 2.1.2 (Minh et al. 2020). We used MODELFINDER to first select the best-fit partitioning scheme and corresponding evolutionary models (Kalyaanamoorthy et al. 2017), and then to infer the best tree and estimate clade support by means of 1000 replicates of ultrafast bootstrapping (Hoang et al. 2018). For Bayesian analyses, the best partition scheme and evolutionary model was first selected with help of the computer program PARTITIONFINDER v2.1.1 (Lanfear et al. 2017). We implemented Bayesian inference with MRBAYES v3.2.6 (Ronquist et al. 2012). The analysis was run for 10 million generations, sampling every 1000, with eight simultaneous Markov Chain Monte Carlo (MCMC) chains, ‘heating temperature’ of 0.15. Support values were calculated as posterior probabilities. We assessed convergence of the chains, correct mixing and the number of burn-in generations with TRACER v. 1.7 (Rambaut et al. 2018). We ran model based analyses remotely at the CIPRES Science Gateway (Miller et al. 2010). The phylogenetic tree was edited for aesthetic purposes using FIGTREE (http://tree.bio.ed.ac.uk/software/figtree/).

### ﻿Species delimitation

We used COI and ITS-2 sequences of a larger sample of Madeiran *Hogna* to explore species boundaries using single marker molecular based approaches. We investigated three alternative methods for species delineation using COI sequences, namely a distance based algorithmic method (Barcode identification number, BIN) (Ratnasingham and Hebert 2013) and two character-explicit methods, one requiring ultrametric trees (General Mixed Yule Coalescent model with single threshold, GMYC) (Fujisawa et al. 2016) and one that does not (multi-rate Poisson tree processes, mPTP) (Kapli et al. 2017). The BIN system was implemented on-line through the BOLD v4 platform (Ratnasingham and Hebert 2007). We inferred gene trees using maximum likelihood following the same strategy specified in the previous section. In addition, we inferred an ultrametric tree using the Bayesian framework for divergence time estimation implemented in BEAST v2.6.3. We assumed a coalescent tree prior (constant population size), which has been suggested to provide a more rigorous test of delimitation since the GMYC model assumes a single species as the null option (Monaghan et al. 2009). We defined the best partition scheme and evolutionary model inferred with PARTITIONFINDER, defined a lognormal relaxed clock and used an informative prior on the mean rate under the uncorrelated lognormal relaxed molecular clock (ucld.mean) parameter derived from the literature (mean = 0.0199, sd. dev.=0.05) (Bidegaray-Batista and Arnedo 2011). Convergence and mixing of MCMC chains were assessed with TRACER v.1.7 (Rambaut et al. 2018). Independent runs were combined with LOGCOMBINER (10% burn-in), and TREEANNOTATOR was used to summarise the information from the sampled trees. The m-PTP model was implemented using a Markov chain Monte Carlo (mcmc) approach, which allows estimates of support values on the delimitations, on the COI matrix. The analyses were conducted on the best IQ-TREE. We ran 5 chains of 100 million generations each, removing the first 2 million as burn-in, and discarding all branches with lengths smaller or equal to 0.0012708187. We used the R package ‘SPLITS’ (Ezard et al. 2017) to fit the GMYC model. Additionally, we estimated haplotype/allele networks for the COI and ITS-2 matrices independently using the statistical parsimony method (Templeton et al. 1992; Clement et al. 2000), with a confidence limit of 95% implemented in the R package ‘HAPLOTYPES’ (Aktas 2015). The ITS-2 sequences were aligned using the phylogeny-aware algorithm implemented in WEBPRANK (Löytynoja and Goldman 2010), specially recommended for aligning closely related sequences. We determined the number of alleles in the ITS-2 matrix considering the gaps as absence/presence data. Uncorrected pairwise genetic distances were calculated in MEGA X (Kumar et al. 2018).

### ﻿Divergence time estimation

In the absence of fossil evidence and to avoid using circular reasoning by using information on the island age, we estimated divergence time using published information on substitution rates in lycosids (Piacentini and Ramírez 2019). We restricted our estimates to the more exhaustively sampled COI gene. Since the COI sequences include both intra and inter-specific relationships, we used a multispecies coalescent (MSC) approach as implemented in STARBEAST2 (Ogilvie et al. 2017), which allows combining coalescent and species (Yule) tree priors. Haplotypes were assigned to species according to the results of the molecular delimitations (see results), which resulted in the combination of *H.insularum* and *H.maderiana* haplotypes in one single lineage. We included sequences of *H.radiata* and *H.ferox* as putative outgroups but did not enforce the root. We assigned unlinked evolutionary models to each codon position, as suggested by PARTITIONFINDER and defined a relaxed lognormal clock with prior rates for the ucld.mean rate as follows: mean = 0.1716 substitutions/mya and Stdev = 0.006. Three independent runs of 50 million generations were performed, sampling every 5000 generations. We assessed convergence and mixing of each MCMC chain and combined them as described above.

### ﻿Morphological analyses

The genus *Hogna*, as shown by Piacentini and Ramírez (2019), is paraphyletic with many of its former species transferred to other genera (Brady 2012). This forbids the elaboration of an identification diagnosis based on the systematic circumscription of the genus. The genus diagnosis created by Dondale and Redner (1990) includes species that were or should probably be placed in other genera for which the only way to identify a species as *Hogna* is to follow the diagnosis provided by Brady (2012). By doing so, we identify the presented species as *Hogna*.

Morphological observations were carried out using a stereomicroscope Leica MZ 16A equipped with a digital camera Leica DFC450. Individual raw photos were taken with the help of the software Leica Application Suite v4.4 and mounted with the software Helicon Focus (Helicon Soft, Ltd.). Further editions were done with Paint Shop Pro v21 (Corel Corporation). The epigyne was removed from female specimens with the aid of hypodermic needles and forceps. To clear the membranous tissues surrounding the spermathecae and copulatory ducts, we manually removed muscular and membranous tissue with forceps and a needle. This process accidentally led to the breakage of some copulatory ducts (usually delicate in the Lycosidae) and cracking of the median septum in some specimens (e.g., Figs 16E, 29B). SEM images of the male copulatory bulb were obtained with a Q-200 (FEI Co.) scanning electron microscope (SEM). For the SEM images, each male pedipalp was excised at the joint between tarsus and tibia. Samples were sonicated for roughly 30 seconds with ultrasonic bath Nahita ZCC001, air dried and carbon or gold sputter-coated. In most cases, the position of the embolus of the SEM samples appears slightly altered (usually directed more anteriorly, closer to the tip of the terminal apophysis) relative to the normal resting position from specimens stored in ethanol. We measured all adult specimens with an ocular micrometre in the stereoscope. All measurements are in millimetres (mm). Description format followed Baert et al. (2008) and genitalic nomenclature followed Langlands and Framenau (2010).

### ﻿Abbreviations

**AW** anterior eye row width;

**Cl** clypeus;

**Fe** femur;

**LMP** length between hind border of posterior eye and front border of median eye;

**MOQ** median ocular quadrangle;

**Mt** metatarsus;

**MW** median eye row width;

**Pa** Patella;

**PW** posterior eye row width;

**Ti** tibia;

**TiIL/D** Length to Diameter of Tibia I.

#### Male genitalia

**AT** apical point;

**C** cymbium;

**E** embolus;

**P** palea;

**R** Ridge;

**TgA** tegular apophysis;

**T** tegulum;

**TmA** terminal apophysis;

**VS** ventral spur.

#### Female genitalia

**AP** anterior pocket;

**MS** median septum;

**PTP** posterior transverse part;

**S** spermatheca;

**D** diverticulum.

#### Collections

**NHM**Natural History Museum, London, UK;

**CRBA** Centre de Recursos de Biodiversitat Animal, University of Barcelona, Barcelona, Spain;

**FMNH** Finnish Museum of Natural History, Helsinki, Finland;

**LCPC** Luís Crespo’s personal collection;

**MIZ** Museum and Institute of Zoology, Polish Academy of Sciences, Warsaw, Poland;

**MMF**Museu Municipal do Funchal, Funchal, Portugal;

**MMUE**Manchester Museum, University of Manchester, Manchester, UK;

**MNHNP** French National Museum of Natural History, Paris, France;

**OUMNH**Oxford University Museum of Natural History, Oxford, UK;

**SMF** Senckenberg Research Institute, Frankfurt am Main, Germany;

**NHRS**Swedish Museum of Natural History, Stockholm, Sweden.

#### Conservation

**AOO** Area of occupancy;

**EOO** Extent of Occurrence.

## ﻿Results

### ﻿Phylogenetic analyses

**Figure 2.**
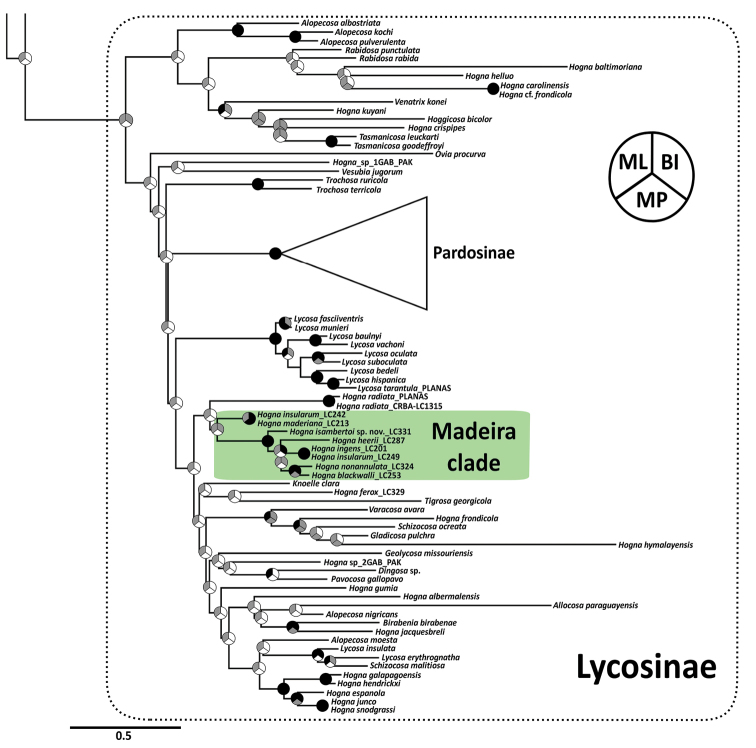
Best maximum likelihood tree of Lycosinae, inferred with IQTREE2 after selecting the best partition scheme and evolutionary models. Nodes are split in three sections, representing the different methods. Support on nodes should be read as follows: black: ML ultrafast bootstrap and BI posterior probability ≥ 0.95, MP Jackknife ≥ 0.7; grey: ML Ultrafast Bootstrap and BI posterior probability < 0.95, MP Jackknife < 0.7; white: unrecovered node.

The concatenated matrix included 2641 characters, 657 bp of the COI, 302 bp H3, and 554 bp of the nad1, and 300 and 828 aligned position for the 12S and 28S, respectively, and 173 terminals including outgroups (see Piacentini and Ramírez 2019). Inferred relationships of the concatenated data matrix are summarised in Fig. 2 (See Suppl. material 3 for full trees for each inference methods). Parsimony analysis of the concatenated data matrix with gaps scored as absence/presence characters resulted in 1,000 trees (overflow) of 16,865 steps. Bayesian maximum clade credibility tree was obtained after removing 40% of the first generations as burn-in. Preferred partition schemes differed between IQTREE2 and PARTITIONFINDER in that the first joined COI and H3 second positions, while the second split by gene and codon position in all cases. Madeiran *Hogna* were recovered as two well-supported clades, one including the species *H.maderiana* and *H.insularum*, hereafter referred as the *maderiana* clade, and the other one including the remaining species, hereafter referred as the *ingens* clade. Model-based analyses inferred the two clades as sister groups, albeit with low support (Fig. 2). Conversely, parsimony inferred the *ingens* clade to be sister to the mainland species *H.radiata*. In all analyses, *H.isambertoi* sp. nov. was supported as sister to the remaining species in the *ingens* clade, while *H.nonannulata* and *H.blackwalli* were supported as sister in model-based analyses. All analyses agreed in supporting a surprisingly close relationship between *H.ingens* and one individual identified as *H.insularum* from Madeira. Similarly, all analyses agreed in showing the genus *Hogna* as a polyphyletic assemblage. Remaining relationships within Lycosoidea including subfamilies, were similar to those reported in Piacentini and Ramírez (2019).

### ﻿Molecular species delimitation

**Figure 3.**
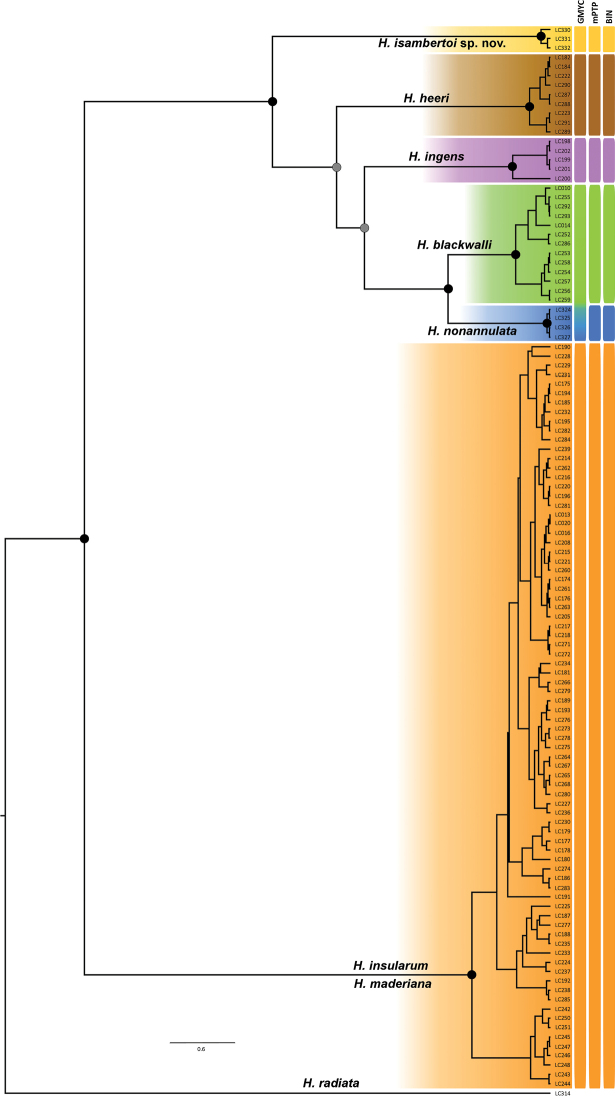
Ultrametric tree for the COI obtained with BEAST using a coalescent (constant population growth) prior to apply the GMYC model. Only unique sequences included. Support on nodes should be read as follows: black: BI posterior probability ≥ 0.95; grey: BI posterior probability < 0.95. Species delimitations based on alternative approaches are indicated with boxes besides the terminal labels.

The COI data matrix included 133 terminals, including a single sequence of the non-Madeiran *Hognaradiata* (Iberian Peninsula), corresponding to 62 haplotypes (one non-Madeiran) (Suppl. material 2). The ITS-2 matrix included 40 terminals with 400 aligned positions and ten additional absence/presence characters, corresponding to 17 alleles (sequence types) (Suppl. material 2). The clustering analysis (BIN) of the COI sequences resulted in six clusters, that mostly matched the morphological circumscription, except for the merging of individuals identified as *H.maderiana* and *H.insularum* (Fig. 3). As already noted in the target multilocus phylogenetic analyses, one individual identified as *H.insularum* (DNA code LC249) clustered together with individuals morphologically assigned to *H.ingens*. Uncorrected genetic distances are shown in Table 1, with unidentified juveniles from the *H.insularum-maderiana* complex listed as “*hx*”. The genetic distance between *H.insularum* and *H.maderiana* was 1.6%, similar to the values observed within *H.insularum* (1.7%). The next lower genetic distance was observed between *H.nonannulata* and *H.maderiana* (4.3%). The largest genetic distances were found between the species pair *H.insularum* and *H.maderiana* and the remaining endemic species (9.9–10.6%) and were similar to those observed with regard to the mainland species *H.radiata* (9.8–11.1%).

**Table 1.** The number of base differences per site from averaging over all sequence pairs within each group are shown. This analysis involved 133 nucleotide sequences. All ambiguous positions were removed for each sequence pair (pairwise deletion option). There was a total of 676 positions in the final dataset. Evolutionary analyses were conducted in MEGA X (Kumar et al. 2018). The presence of n/c in the results denotes cases in which it was not possible to estimate evolutionary distances. hins_mad_LC336_5 and hins_ma_LC249_5 are included in *H.ingens*. *Hx* refers to non-identified juveniles. Grey cells refer to comparison with a continental taxon, yellow cells refer to comparison within the *ingens* clade, and green cells refer to comparison within the *maderiana* clade.

	*radiata*	*heeri*	*ingens*	*nonannulata*	*blackwalli*	*isambertoi*	*maderiana*	*insularum*	*hx*
*radiata*									
*heeri*	0.105	**0.006**							
*ingens*	0.111	0.065	**0.004**						
*nonnanulata*	0.106	0.059	0.064	**0.009**					
*blackwalli*	0.098	0.074	0.073	0.043	**0**				
*isambertoi*	0.107	0.07	0.082	0.075	0.084	**0.003**			
*maderiana*	0.103	0.106	0.105	0.103	0.105	0.095	**0.007**		
*insularum*	0.102	0.104	0.108	0.099	0.104	0.098	0.016	**0.017**	
*hx*	0.103	0.106	0.108	0.103	0.105	0.098	0.01	0.016	**0.01**

The mPTP analysis ran on the IQ-TREE inferred tree, recovered the same groupings with high support. The GMYC model delimited five groups, by merging *H.nonannulata* and *H.blackwalli* together, but the likelihood ratio test revealed that it did not provide a significantly better fit than the null model (one single species, p = 0.7764125).

**Figure 4.**
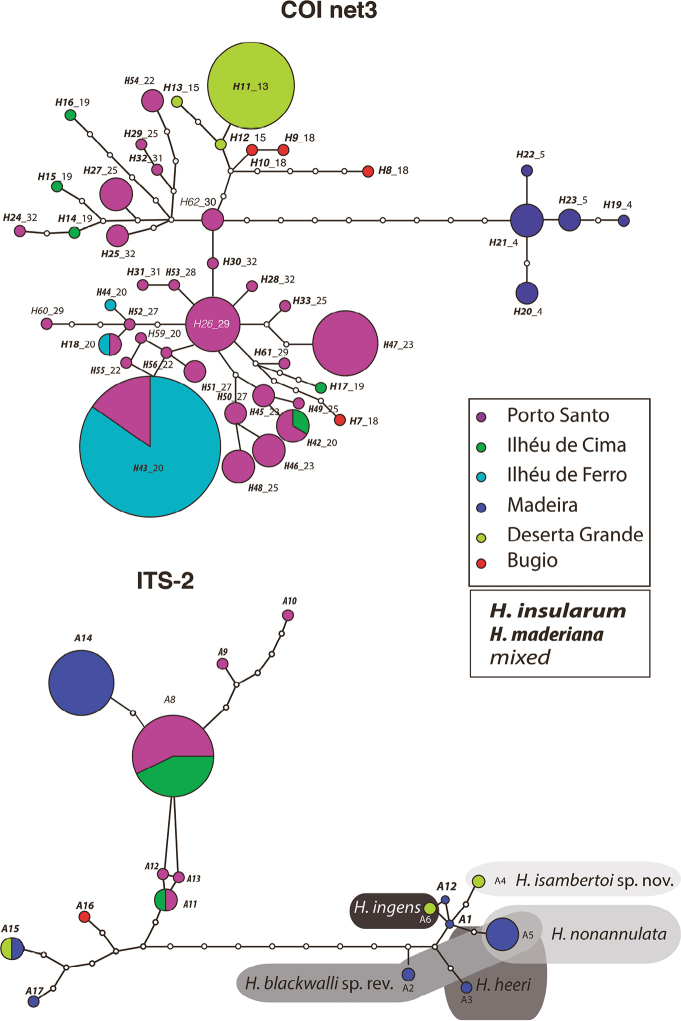
COI haplotype (upper) and ITS-2 allele (lower) networks inferred under statistical parsimony (0.95 probability). Pie size proportional to number of individuals which exhibited the same haplotype/alleles. White circles represent missing haplotypes/alleles. Colours correspond to islands (colour codes in upper box). For the COI haplotypes only the network (3) including *H.insularum* / *H.maderiana* haplotypes showed (each remaining nominal species were resolved as independent networks). ITS-2 alleles boxed per species, except for *H.insularum* / *H.maderiana*. Haplotype/allele labels for *H.insularum* in bold and italics, *H.maderiana* in condensed bold and italics, not assigned in light italics (see lower box legend).

The statistical parsimony analysis at 95% connection resulted in six independent networks that exactly matched the BIN and mPTP clusters (Fig. 4). Lowering the connection limited to 90% had no effect on the results. For the ITS alleles, a single network was obtained (both at 90%and 95%). The alleles of the species *H.maderiana* and *H.insularum* were mixed up, while the rest of alleles were exclusive to each species, except for *H.heeri*, *H.blackwalli* and *H.nonannulata* that shared one allele. The alleles of the putative *H.insularum* individuals bearing *H.ingens*COI haplotypes, were also observed to cluster close to the *H.ingens* alleles.

### ﻿Divergence time estimation

**Figure 5.**
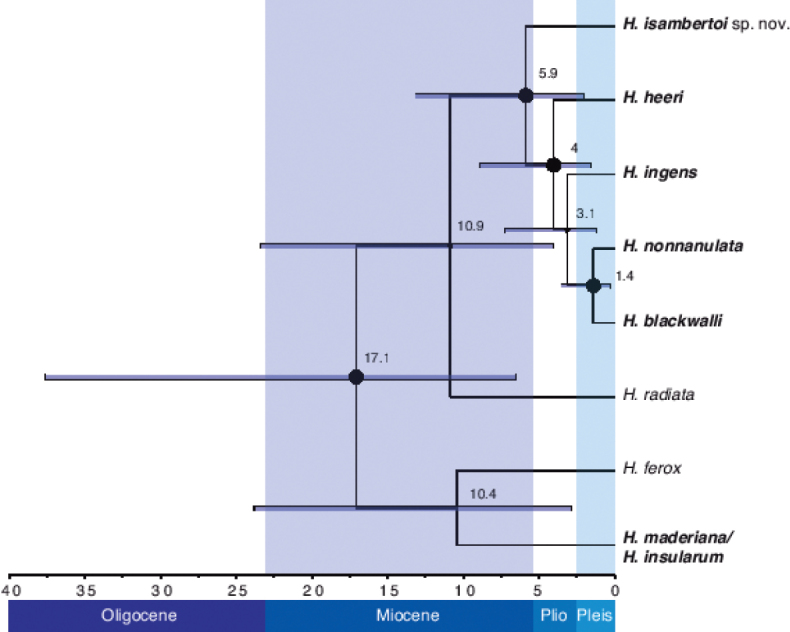
Species tree for the Madeiran *Hogna* including two outgroups. Values on nodes are estimated divergence times in millions of years (my). Dots on nodes indicate BI posterior probability >0.95. Bars correspond to the 95%HPD of the time estimates.

The inferred species tree suggested non-monophyly of Madeiran *Hogna* albeit with low support (Fig. 5). Estimated time of split from their closest sister taxa was similar for the two Madeiran lineages (10.9 mya, 4–23 mya 95%HPD, and 10.4 mya, 2.8–24 mya, for the *ingens* and the *maderiana* clades, respectively). The most recent common ancestor of the *ingens* clade was 5.9 mya (2–13.1 mya). The coalescent times inferred from the COI tree for the different species were 0.09 mya for *H.isambertoi* sp. nov., 0.13 for *H.heeri*, 0.26 for *H.ingens*, 0.4 for *H.blackwalli* and 0.06 for *H.nonannulata*, and 1.17 for the *maderiana* clade.

### ﻿Taxonomy

#### Family Lycosidae Sundevall, 1833

##### ﻿Genus Hogna

Taxon classificationAnimaliaAraneaeLycosidae

Simon, 1885

A8027253-1B45-580D-84FD-1B4E552B79AC

###### Type species.

*Hognaradiata* (Latreille, 1817).

###### Diagnosis.

We follow the diagnosis presented by Brady (2012).

##### ﻿ Hogna blackwalli

Taxon classificationAnimaliaAraneaeLycosidae

(Johnson, 1863)

39F12636-8644-5F24-B82E-FA76BF117B10

Figures 6 , 7 , 8

Lycosa blackwalli Johnson, 1863: 152 (Dmf).
Trochosa maderiana Thorell, 1875: 167 (mf, misidentification).
Geolycosa blackwalli Roewer, 1955: 241.
Geolycosa blackwalli Roewer, 1960: 691, fig. 387a–d (mf).
Geolycosa ingens Denis, 1962: 96, f. 78 (f, misidentification).
Hogna maderiana Wunderlich, 1992: 461, fig. 720c–e (mf, S).
Hogna maderiana Wunderlich, 1995: 416, fig. 28 (f).

###### Types.

***Syntypes***: Madeira • 2 ♀♀; Pico Ruivo, leg. Johnson, stored at OUMNH, collection number 1617. Examined.

###### Material examined.

Madeira • between Pico do Areeiro and Poiso, 1 ♀ (SMF65685), leg. K. Groh; Caramujo, 32.77161°N, 17.06205°W, 1 ♂ (CRBALC0010: LC010), 23.VIII.2016 (collected as subadult, reared in captivity to adult on 7.X.2016), hand collecting, leg. L. Crespo; “Funchal” [probably north of it because “600 to 2000 ft.” is written in label], 1 ♀ (NHM, mounted dry), V.1895, leg. O. Grant; Paúl da Serra, 1 ♀ (SMF65684), hand collecting, leg. I. Silva, 1 ♀ (CRBALC0496: LC254) and 3 juveniles (CRBALC0495: LC253, CRBALC0497: LC255, CRBALC0499: LC256), 32.78182°N, 17.09978°W, 28.III.2017, hand collecting, leg. L. Crespo & I. Silva; Paúl da Serra / Rabaçal, 5 ♀♀ (SMF65696); Pico do Areeiro, 32.739067°N, 16.934448°W, 1 ♀ (CRBALC0516: LC270), 27.III.2017, hand collecting, leg. I. Silva; Pico do Cidrão, 32.74036°N, 16.93877°W, 1 ♀ (CRBALC0489: LC286), 27.III.2017, hand collecting, leg. L. Crespo; Rabaçal, 1 ♀ (MNHNP AR16185), IV.1957, leg. H. Coiffait, 1 ♀ (SMF65683), 18.VIII.1991, hand collecting, leg. I. Silva; Ribeiro Bonito, 32.79582°N, 16.93710°W, 1 juvenile (CRBALC0014: LC014), 4.VIII.2016, hand collecting, leg. L. Crespo; trail from Paúl da Serra to Montado dos Pessegueiros, 32.78837°N, 17.09857°W, 1 ♀ (CRBALC0271: LC252) and 2 juveniles (CRBALC0498: LC292, CRBALC0502: LC293), 28.III.2017, hand collecting, leg. L. Crespo & I. Silva, 2 ♀♀ (CRBALC0503: LC257, CRBALC0515: LC259) and 1 juvenile (CRBALC0514: LC258), 31.III.2017, hand collecting, leg. L. Crespo, M. Arnedo & P. Oromí, 1 ♂ (CRBALC0718), 2 ♀♀ (CRBALC0601, CRBALC0605) and 2 juveniles (CRBALC0603, CRBALC0698), 4.IV.2018, hand collecting, leg. L. Crespo & A. Bellvert; 1 ♀ (SMF9910750), 1 ♂, 2 ♀♀ and 4 juveniles (NHRS-JUST-000001114), 2 ♀♀ (NHM, mounted dry), [no collection data except for the data of collection of one of these females, IX.1963].

###### Diagnosis.

**Figure 6.**
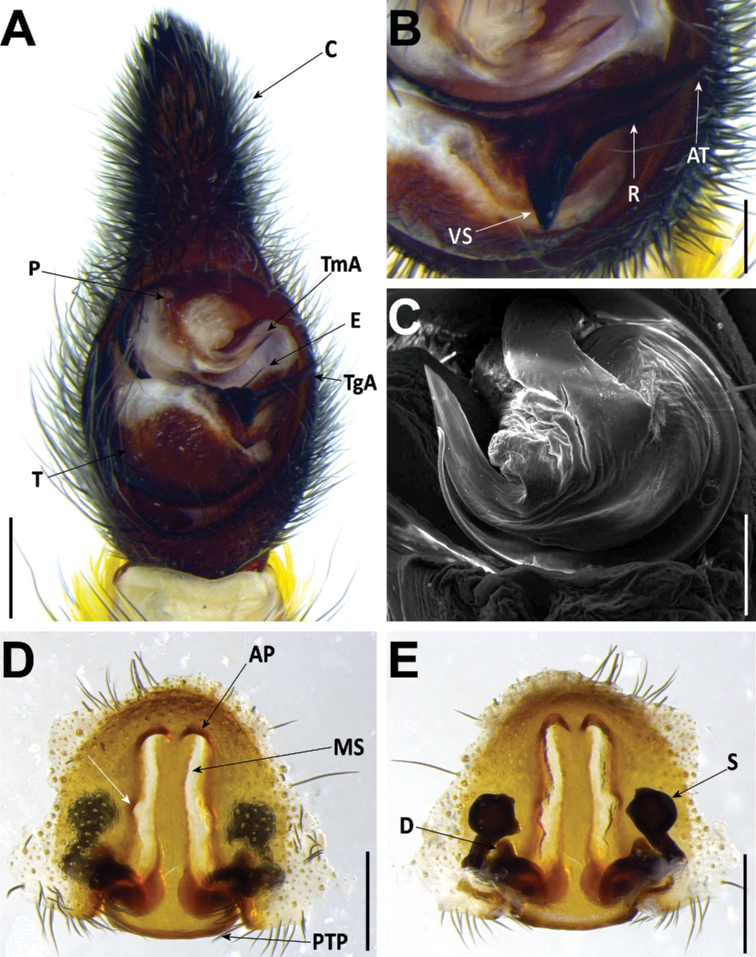
*Hognablackwalli***A–C** male (CRBALC0718): **A** left male pedipalp, ventral **B** detail of the median apophysis, anteroventral **C**SEM image, right male pedipalp, ventral **D, E** female (CRBALC0516): **D** epigyne, ventral (white arrow points to an indentation that may be helpful for diagnosis) **E** vulva, dorsal. Abbreviations, male pedipalp: AT – anterior point, C – cymbium, E – embolus, P – palea, R – ridge, T – tegulum, TA – terminal apophysis, TgA – tegular apophysis, VS – ventral spur. Abbreviations, female genitalia: D – diverticulum, H – epigynal hoods, MS – median septum, S – spermatheca. Scale bars: 0.5 mm (**A, D, E**); 0.2 mm (**B, C**).

*Hognablackwalli* can be diagnosed from all other Madeiran *Hogna* by the aspect of its legs, with two small patches of yellow setae in the joints of anterior tibiae with metatarsi and of metatarsi with tarsi (Fig. 26A). In addition, by the genitalia: in males, the embolus with tip tilted retrolaterally and a tegular apophysis with a long, sharp ventral spur (Fig. 6A–C). In females, the epigynal anterior pocket shows a small indentation on the lateral border (white arrow in Fig. 6D).

###### Redescription.

**Male** (*CRBALC0718*): (Fig. 6A–C). Total length: 18.9; carapace: 9.1 long, 6.8 wide.

***Colour***: carapace brown, with short black setae except anteriorly and laterally, where short white setae and long black setae are present; median cream longitudinal band present, covered with short white setae, anteriorly broadened, with suffused greyish brown patches covered by yellow setae; two yellow marginal bands, suffused with greyish brown patches, covered with short white setae; four black striae well visible on each flank. Chelicerae black, covered mostly in black setae but with sparse yellow setae. Gnathocoxae very dark orange-brown, labium blackish; sternum black, with a faint, thin longitudinal stripe extending to less than half of sternum length. Legs grey to greyish brown, with seven or eight patches of white setae (anterior legs with eight, posterior legs seven) except the patches in anterior metatarsi, both yellow. Pedipalpal femur as legs, patella, tibia and proximal cymbium with yellow to orange setae, apical cymbium covered in black setae. Abdomen with a pair of anterolateral black patches, extending laterally into grey to black flanks, interspersed with white patches; a median orange lanceolate patch is bordered by the aforementioned pattern, posteriorly also by dark chevrons; venter with a wide longitudinal black band, bordered by a mesh of white and black patches.

***Eyes***: MOQ: MW = 0.7 PW, MW = 1.1 LMP, MW = 1.1 AW; Cl = 0.5 DAME. Anterior eye row slightly procurved.

***Legs***: Measurements: Leg I: 27.3, Ti: 6.4; Leg IV: 29.7, Ti: 6.6; TiIL/D: 5.8. Spination of Leg I: Fe: d1.1.0, p0.0.2; Ti: p0.0.1, v2l.2l.2s; Mt: p0.0.1, r0.0.1, v2l.2l.1s. Mt with very dense scopulae.

***Pedipalp***: cymbium with eight dark, stout, macrosetae at tip, Fe with two dorsal and an apical row of four spines, Pa with one prolateral spine, Ti with one dorsal, one dorsoprolateral, and one prolateral spines. Tegular apophysis with ventral spur long, sharp, with a concave ridge leading to a thin apical point (Fig. 6A, B); terminal apophysis blade-shaped with sharp end (Fig. 6A–C); embolus short, with tip directed laterally (Fig. 6A–C); palea large (Fig. 6A).

**Female** (*CRBALC0516*): (Fig. 6D, E). Total length 29.9; carapace: 10.4 long, 8.0 wide.

***Colour***: overall as in male, but darker. Sternum entirely black. Yellow setae in pedipalp restricted to the joints of tibia with tarsus and patella with tibia.

***Eyes***: MOQ: MW = 0.7 PW, MW = 1.2 LMP, MW = 1.1 AW; Cl = 0.7 DAME. Anterior eye row slightly procurved.

***Legs***: Measurements: Leg I: 27.7, TiI: 6.3; Leg IV: 31.8, TiIV: 6.8; TiIL/D: 3.8. Spination of Leg I: FeI: d1.1.0, p0.0.2; TiI: p0.0.1, v2l.2l.2s; MtI: p0.0.1, r0.0.1, v2l.2l.1s. MtI with very dense scopulae.

***Epigyne***: anterior pockets almost touching, short, with lateral borders anteriorly parallel, medially slightly divergent after a small sinuosity (white arrow in Fig. 6D); anterior pocket cavities deep; median septum with narrow posterior transverse part (Fig. 6D); spermathecae globular (Fig. 6E); copulatory ducts with small, stout diverticulum ventrally (Fig. 6E); fertilisation ducts emerging at the base of copulatory duct (Fig. 6E).

**Figure 7.**
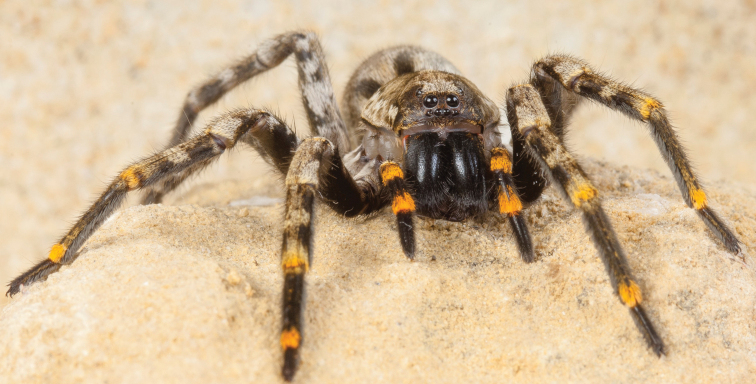
Photograph of *H.blackwalli*. Female specimen, recently dead, in captivity. Photograph credit Emídio Machado.

###### Intraspecific variation.

Carapace length, males: 7.4–9.1, females: 8.9–10.4. Suffused greyish brown patches in median yellow longitudinal band not necessarily covered with yellow setae. Epigyne can present two small depressions in the base of median septum, which can be of variable length, position and concavity of inflexion of the lateral hood walls can also be variable, either placed near hoods or medially, median septum can be swollen medially.

###### Distribution.

**Figure 8.**
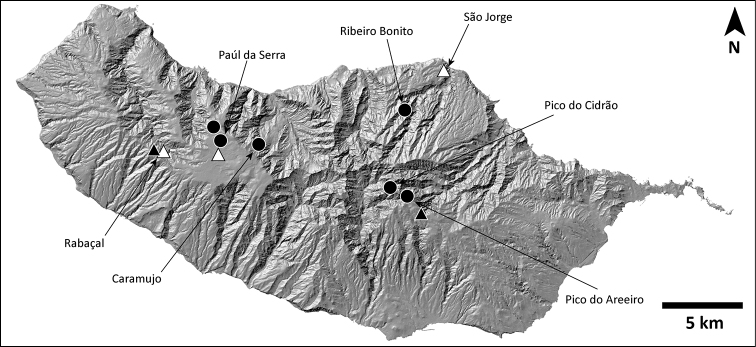
Distribution of *H.blackwalli*. Circles: present records; black triangles: revised records from literature; white triangles: unconfirmed records from literature.

This species is known from areas in or near the laurel forest patch in Madeira, in the north half of the island (Fig. 8).

###### Ecology.

*Hognablackwalli* can be found in montane grasslands surrounding laurel forest areas or *Erica* shrubland. Surprisingly, it can also be found in closed canopy laurel forest, where, at night, specimens can be found climbing tree trunks.

###### Conservation status.

*Hognablackwalli* was assessed according to the IUCN Red List criteria as *H.maderiana*, with the status of Least Concern (Cardoso et al. 2018a). The coastal records reported in the referred publication are probably of *H.nonannulata*.

###### Comments.

There has been a great deal of confusion surrounding *H.blackwalli* and *H.maderiana*. Walckenaer’s original description of *H.maderiana* (Walckenaer 1837) based on material from Madeira island indicated that legs were “(…) reddish brown, suffused brown underneath (…)”. Subsequently, Blackwall described the alleged male of Walckenaer’s *H.maderiana* but mentioned a striking leg coloration: “(…) the femora, on the upper side, have a yellowish grey hue, that of the tibia, metatarsi and tarsi being bright orange-red, and the colour of the underside of all the joints is dark brown tinged with grey; (…)” (Blackwall 1857). Additionally, he reported the locality of origin of those specimens to be Porto Santo, not Madeira. Six years later, Johnson (1863) described *H.blackwalli* from Madeira island, indicating that “The metatarsus and tarsus of the two anterior pairs of legs are black, or very dark brown. At the distal extremities and on the upper sides of the femur and genua of the first two pairs of legs, as well as at the extremities of some of the joints of the two posterior pairs of legs, there is a patch of orange setae”. In the same publication, he also described and identified as *H.maderiana* specimens from Ilhéu de Ferro, near Porto Santo. It is unclear on how many specimens Johnson based his description, but we could locate a part of this material at the OUMNH, thus revalidating *H.blackwalli* Johnson, 1863.

The next author to make a taxonomic contribution on these spiders was Thorell (1875), who redescribed *H.maderiana* based on specimens from Madeira. However, his reference to the legs colouration that reads “palporum partibus pateliari et tibiali apice supra croceis, metatarsis tibiisque pedum anteriorum apice quoque croceis vel flavis” suggests that his redescription corresponds to *H.blackwalli* instead. We could locate 14 specimens labelled as *H.maderiana* in the NHRS, which most likely were the ones examined by Thorell, and we confirmed they correspond to *H.blackwalli*. Kulczynski (1899) followed Blackwall’s judgement to redescribe the large specimens from Porto Santo and Ilhéu de Ferro under the name *Trochosamaderiana*. Almost one century later, Roewer (1960) provided redescriptions of three Madeiran *Hogna*, but no reference was given to the leg coloration, which is the easiest way to distinguish these larger, aforementioned species. His epigyne drawings provided little additional information and were confusing. While the epigyne of *H.ingens* allows identification of this species (Roewer 1960: fig. 387e), the same is not true for the illustrations of *Isohognamaderiana* and *Geolycosablackwalli* (Roewer 1960: figs 319a and 387a, respectively), which look rather the same. However, he reports that Thorell’s *Trochosamaderiana* specimens are *H.blackwalli*, for which we assume Roewer’s redescription of *Geolycosablackwalli* to correspond to the same species we identify as *H.blackwalli*. Denis (1962) cited two females of *Geolycosaingens* (Blackwall, 1857) and one male and two females of *H.insularum* from locations where *H.blackwalli* is usually found, Rabaçal and Paúl da Serra, on Madeira island. We could find the female identified as *H.insularum* (MNHNP AR16185), and confirm that this is *H.blackwalli*. We confidently attribute the remaining citations of *H.insularum* (specimens not found) to misidentified specimens of *H.blackwalli* The last taxonomic works on Madeiran *Hogna* were by Wunderlich (1992, 1995). In the first of these (Wunderlich 1992), the species *H.maderiana* and *H.blackwalli* were wrongly synonymised and it was stated that “up to Denis (1962), most authors assumed that *H.maderiana* occurred both in Madeira and Porto Santo.” This is not accurate, since Johnson discriminated between *H.blackwalli* from Madeira and *H.maderiana* from Ilhéu de Ferro. In fact, this synonymy is even stranger because while revising the material present at the SMF, we found vial 9910750 of the Roewer collection, with an identification note by Wunderlich stating “*H.blackwalli* (Johnson)”. Finally, we have located only part of the type material described by Johnson at the OUMNH, because no males were found, even though his description mentioned males. Therefore, the whereabouts of the remaining specimens of the type series are unknown.

##### ﻿ Hogna ferox

Taxon classificationAnimaliaAraneaeLycosidae

(Lucas, 1838)

F0BBB71B-E93B-59C3-8466-92B780654F18

Arctosa maderana Roewer, 1960: 604–605, fig. 334a (f), fig. 334 b (m). Syn. nov. (see WSC 2021 for a complete list of synonymies)

###### Types.

***Holotype***: 1 ♀ (with 1 paratype ♂ in vial), leg. Roewer, stored at SMF, collection number 9903912. Examined.

###### Material examined.

Gran Canaria • Gando, 1 ♂ (SMF25851), X.1961, leg. G. Shmidt; La Rosetas, 28.12196°N, 15.68662°W, 4 ♀♀ (CRBALC0586, CRBALC0602: LC329, CRBALC0706, CRBALC0719), 21.IV.2018, leg. L. Crespo & A. Bellvert; Playa del Inglés, 1 ♀ (SMF25422), 1970, leg. G. Schmidt; San Sebastian, 1 ♀ (SMF29107), IV.1974, leg. G. Schmidt. La Gomera • Lomada near San Sebastian, 1 ♀ (SMF29134), IV.1974, leg. Wild. Tenerife • La Orotava, 28.36666°N, 16.51666°W, 1 ♂ (SMF2234), 1871, leg. Grenacher & Noll. Tunisia • Jendouba, 1 ♀ (SMF63576), X.1995, leg. G. Eichler; (no sampling information), 1 ♀ (SMF37118). (No country or sampling information) • 2 ♂♂, 1 ♀ and 1 juvenile (SMF67996).

###### Justification of the synonymy.

After its original description, the endemic species *Arctosamaderana* Roewer, 1960 was never again recorded in the archipelago of Madeira, despite extensive sampling through several biodiversity inventory projects (Crespo et al. 2014a; Boieiro et al. 2018; Malumbres-Olarte et al. 2020). We identified the type female and the paratype male as *H.ferox* (Lucas, 1838). *Hognaferox* has a widespread distribution throughout the Mediterranean, being present in the Iberian Peninsula, North Africa, and the neighbouring archipelago of the Canary Islands. However, it has never been reported in Madeira, and after examination of specimens, we propose that *A.maderana* Roewer, 1960 is a junior synonym of *H.ferox* (Lucas, 1838) and should be removed from the Madeira archipelago fauna.

##### ﻿ Hogna heeri

Taxon classificationAnimaliaAraneaeLycosidae

(Thorell, 1875)

2BFF3432-5024-5B6E-B7C5-A338BDB4ED8A

Figures 9 , 10 , 11

Trochosa herii Thorell, 1875: 166 (Df).
Trochosa herii Kulczynski, 1899: 433, pl. 9, fig. 188 (f).
Hogna heeri Roewer, 1955: 248.
Hogna herii Roewer, 1959: 411, fig. 221a–d (f, Dm).
Hogna heeri Wunderlich, 1992: 459, fig. 720, 720a (mf).

###### Types.

***Syntypes***: Madeira • 2 ♀♀, leg. O. Heer, stored at NHRS, collection number JUST-000001113. Examined.

###### Material examined.

Bugio • Planalto Sul, 32.41228°N, 16.47466°W, 1 ♀ (LCPC), 3.XII.2012, hand collecting, leg. I. Silva. Madeira • between Eira do Serrado and Curral das Freiras, 1 ♀ (SMF69107); Paúl da Serra, 2 ♀♀ (MMUE G7572.874), 25.IV.1973, leg. J. Murphy, 1 ♀ (CRBALC0492: LC289), 32.78182°N, 17.09978°W, 19.III.2017, hand collecting, leg. I. Silva, 1 ♀ (CRBALC0500: LC222) and 1 juvenile (CRBALC0494: LC291), 28.III.2017, leg. I. Silva; Pico do Cidrão, 32.74036°N, 16.93877°W, 1 ♀ (LCPC), 24.VI.2003, pitfall trapping, leg. M. Freitas, 2 ♀♀ (CRBALC0490: LC287, CRBALC0288: LC288), 27.III.2017, hand collecting, leg. L. Crespo & I. Silva; trail from Paúl da Serra to Montado dos Pessegueiros, 32.78837°N, 17.09857°W, 2 ♀♀ (CRBALC0270: LC184, CRBALC0501: LC223) and 1 juvenile (CRBALC0493: LC290), 28.III.2017, hand collecting, leg. L. Crespo & I. Silva; 1 ♀ (SMF37575).

###### Diagnosis.

**Figure 9.**
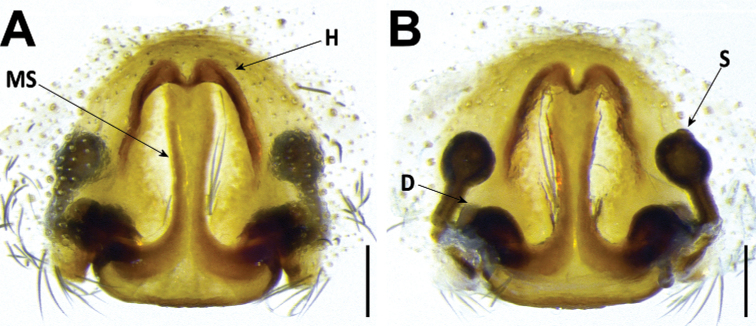
*Hognaheeri* female (CRBALC0501): **A** epigyne, ventral **B** vulva, dorsal. Abbreviations, female genitalia: D – diverticulum, H – epigynal hoods, MS – median septum, S – spermatheca. Scale bars: 0.2 mm.

*Hognaheeri* can be diagnosed by the genitalia: the males, according to literature, by a straight embolus (Wunderlich 1992: 595, fig. 720); in females, by epigynal anterior pockets with widely divergent lateral border and median septum with a wide posterior transverse part (Fig. 9). Similar species include *H.insularum* and *H.isambertoi* sp. nov., from which it cannot be somatically differentiated.

###### Redescription.

**Male**: We could not examine any male specimens.

**Female** (*CRBALC0500*): (Fig. 7 corresponds to specimen CRBALC0501). Total length 13.54; carapace: 5.63 long, 4.4 wide.

***Colour***: carapace greyish brown, covered with short black setae, with a median cream longitudinal band, anteriorly broadened, covered with short white setae, with suffused greyish brown patches; two yellow marginal bands, with roughly round grey patches, covered with short white setae; four black striae well visible on each flank. Chelicerae dark brown, covered in black and yellow setae. Gnathocoxae and labium overall brown, with posterior margin blackish; sternum yellow, with a faint V-shaped grey patch and grey lateral borders. Legs yellow, with irregular grey suffused patches, except metatarsi and tarsi, brown. Pedipalps yellow except tibia, brown, tarsus, blackish brown. Abdomen with a pair of anterolateral black patches, extending laterally into grey flanks, mottled with yellowish patches covered with white setae; a median dark lanceolate patch is bordered by two yellowish longitudinal bands interconnected in anterior half, posteriorly by means of dark chevrons; venter yellowish, with a median dark grey longitudinal band, bordered by yellowish and grey small patches.

***Eyes***: MOQ: MW = 0.7 PW, MW = 1.1 LMP, MW = 1.1 AW; Cl = 0.9 DAME. Anterior eye row straight.

***Legs***: Measurements: Leg I: 13.0, TiI: 2.8; Leg IV: 16.10, TiIV: 3.22; TiIL/D: 3.7. Spination of Leg I: FeI: d1.1.1, p0.0.1; TiI: v2l.2l.2s; MtI: p0.0.1, r0.0.1, v2l.2l.1s. MtI with sparse scopulae in basal half and dense scopulae on distal half.

***Epigyne***: anterior pockets touching, short, with lateral borders widely divergent, converging solely at its posterior end (Fig. 9A); anterior pocket cavities deep; median septum with wide posterior transverse part (Fig. 9A); spermathecae globular (Fig. 9B); copulatory ducts basally with a laterally projected diverticulum (Fig. 9B); fertilisation ducts emerging at the base of copulatory duct (Fig. 9B).

**Figure 10.**
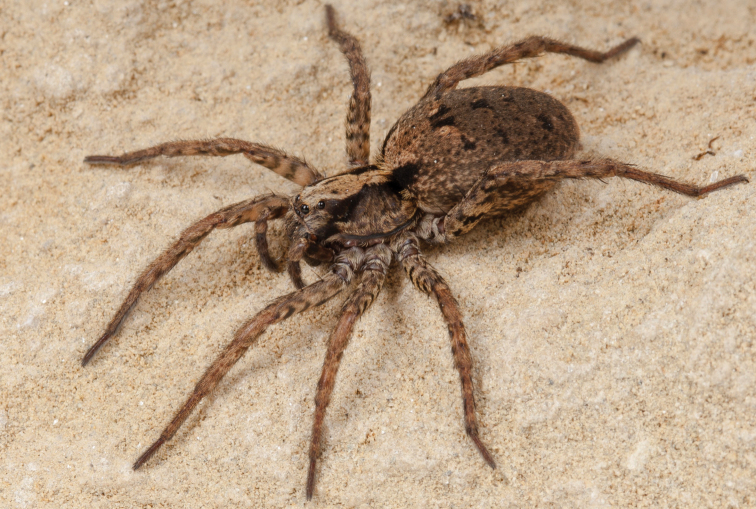
Photograph of *H.heeri*. Female specimen in captivity. Photograph credit Emídio Machado.

###### Intraspecific variation.

Carapace length, females: 5.6–5.8. In females, the ventral abdominal dark band may be entirely absent; the relative position of female epigynal anterior pockets may vary from touching to almost touching.

###### Distribution.

**Figure 11.**
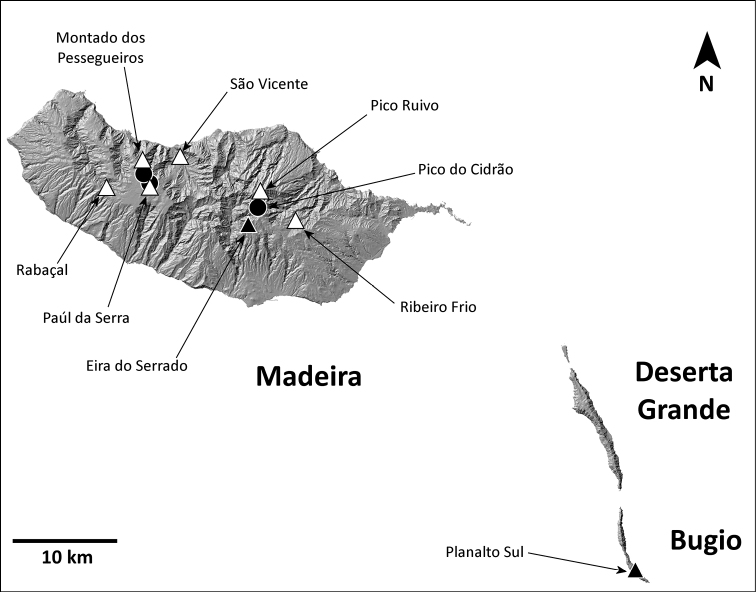
Distribution of *H.heeri*. Circles: present records; black triangles: revised records from literature; white triangles: unconfirmed records from literature.

This species is known from two distinct regions: high altitude localities in Madeira, always above 800 m, and the island of Bugio (Fig. 11).

###### Ecology.

*Hognaheeri* occurs in montane grasslands or *Erica* shrubland in Madeira and the steep, semi-arid summit of Bugio.

###### Conservation status.

*Hognaheeri* was assessed according to the IUCN Red List criteria, with the status of Least Concern (Cardoso et al 2018b).

###### Comments.

The specific epithet of *H.heeri* has been one of the names renamed by Bonnet (1959), who changed all previously described spider species’ names which were patronyms ending in “ii” to end in “i”, as a way to correct spelling (Bonnet 1945). Although the ICZN argues for the maintenance of the original spelling, common usage dictates that these modified spellings continue to be used. The disjunct distribution of *H.heeri*, with populations in Madeira and Bugio, is somewhat baffling. The only known specimens from Bugio previously reported (Crespo et al. 2013) were examined: while the female matches *H.heeri*, the male pedipalp is the same as that of *H.isambertoi* sp. nov., with the tip of the embolus slightly tilted anteriorly (Fig. 18A). We would like to remark that Wunderlich reported an apophysis at the base of the embolus (indicated with an arrow in his figure) as a diagnostic feature to identify males of *H.heeri* (Wunderlich 1992: fig. 720), which appears to be either inconspicuous or missing altogether. To us it seems the arrow is pointing to the pars pendula membrane connecting the terminal apophysis with the embolus. Unfortunately, we could not gather molecular information from the Bugio specimens due to their poor preservation. Lastly, while revising Thorell’s type series, we identified one of the three adult females in the original vial as *H.insularum*.

##### ﻿ Hogna ingens

Taxon classificationAnimaliaAraneaeLycosidae

(Blackwall, 1857)

3DAD3143-0A20-5828-B491-DA3E0127F7CE

**Figure 12.**
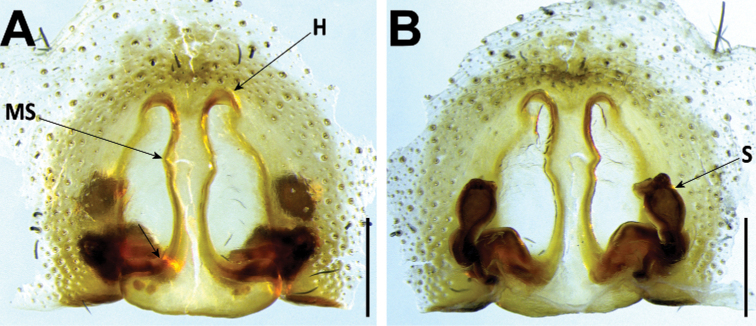
*Hognaingens* female (CRBALC0591): **A** epigyne, ventral **B** vulva, dorsal. Abbreviations, female genitalia: H – epigynal hoods, MS – median septum, S – spermatheca. Scale bars: 0.5 mm.

**Figure 13.**
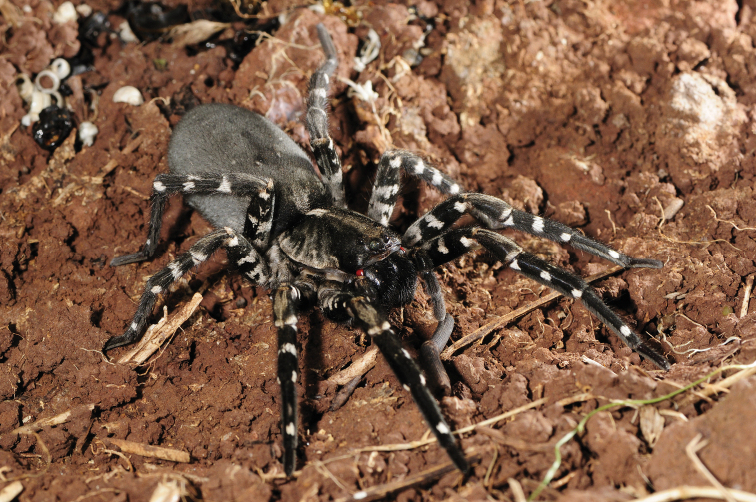
Photograph of *H.ingens*. Female specimen in the field. Photograph credit Pedro Cardoso.

**Figure 14.**
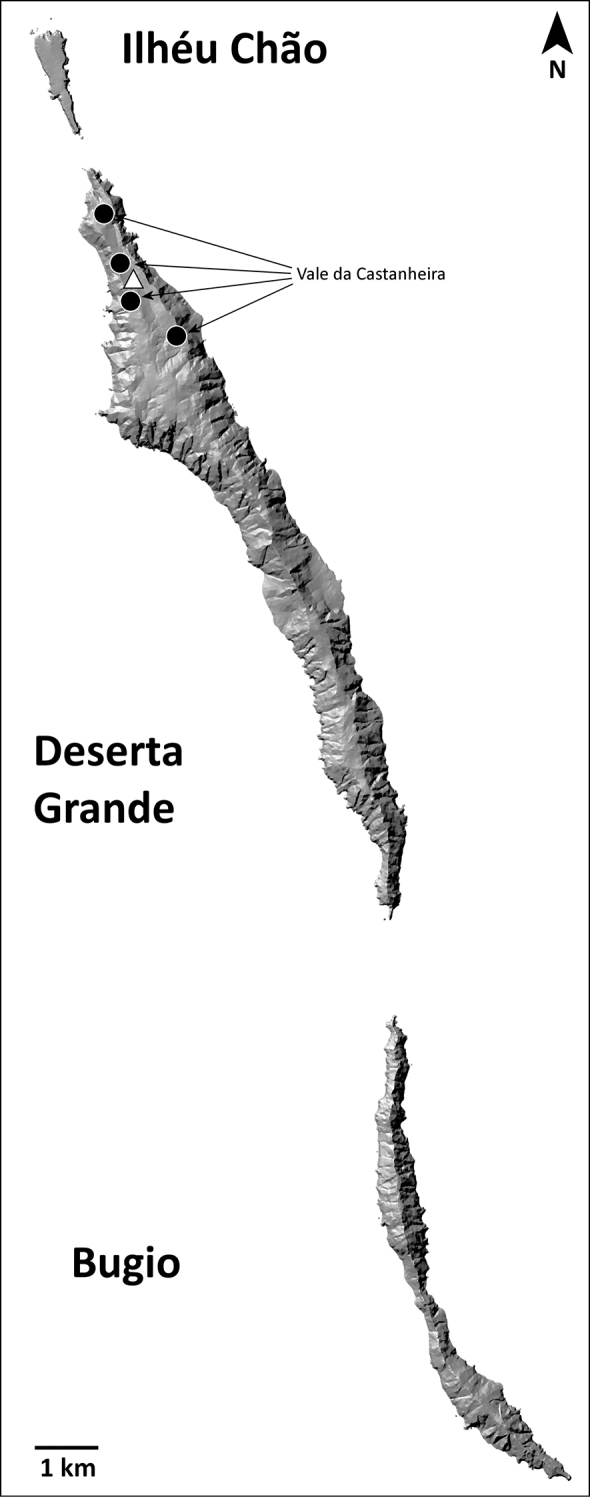
Distribution of *H.ingens*. Black circles: present records; white triangle: unconfirmed record from literature.

Figures 12 , 13 , 14

Lycosa ingens Blackwall, 1857: 284 (Df).
Lycosa ingens Blackwalli, 1867: 203 (Dm).
Trochosa ingens Kulczynski, 1899: 423, pl. 9, fig. 121 (mf).
Geolycosa ingens Roewer, 1955: 241.
Geolycosa ingens Roewer, 1960: 689, fig. 387e (f).
Hogna ingens Wunderlich, 1992: 459, fig. 720b, fig. 724a.

###### Types.

***Holotype***: no type materials from the Blackwall collection were found neither at the OUMNH nor the NHM.

###### Material examined.

Deserta Grande • Vale da Castanheira (N), 1 ♀ (SMF21994), 26.III.1967, 1 ♀ (CRBALC0591) and 4 juveniles (CRBALC0593, CRBALC0594, CRBALC0595, CRBALC0592), 32.56685°N, 16.53694°W, 25.III.2017, hand collecting, leg. L. Crespo; (unknown location), 3 ♀♀ (MNHNP AR16186).

###### Diagnosis.

*Hognaingens* can be diagnosed from all other Madeiran *Hogna* by the aspect of its legs, blackish with white patches (Figs 13, 26C), and additionally by its genitalia. In males, according to literature, by the inclined palea shield (Wunderlich 1992: 596, fig. 720f). In females, by short epigynal anterior pockets, with lateral borders divergent and anteriorly swollen median septum (Fig. 12A).

###### Redescription.

**Male**: We could not examine any male specimens.

**Female** (*CRBALC0591*): (Fig. 12). Total length 25.1; carapace: 14.8 long, 11.0 wide.

***Colour***: carapace greyish brown, densely covered with short black setae, with a cream longitudinal band present from fovea to posterior margin of carapace; with two faint light grey marginal bands suffused with black patches, covered with white setae; four striae well visible on each flank. Chelicerae black except apically, reddish brown, covered in black setae. Gnathocoxae and labium overall orange-brown, densely covered with black setae; sternum greyish brown, densely covered with black setae. Legs greyish, with a variable number (6–8) of lightly coloured patches covered by white setae. Pedipalps greyish, densely covered in black setae. Abdomen densely covered in black setae, with only four very small white patches dorsally and a small anterolateral band of white setae; venter densely covered in black setae, with only two faint median bands of small white patches.

***Eyes***: MOQ: MW = 0.7 PW, MW = 1.2 LMP, MW = 1.1 AW; Cl = 0.5 DAME. Anterior eye row slightly procurved.

***Legs***: Measurements: Leg I: 37.7, TiI: 8.9; Leg IV: 35.9, TiIV: 8.4; TiIL/D: 2.3. Spination of Leg I: FeI: d1.1.0, p0.0.2; TiI: p0.0.0, v2s.2s.2s; MtI: p0.0.1, r0.0.1, v2s.2s.1s. MtI an TiI with dense scopulae.

***Epigyne***: anterior pockets far apart, short, with lateral borders anteriorly convergent, then becoming divergent (Fig. 12A); anterior pocket cavities shallow; median septum anteriorly swollen, with wide posterior transverse part (Fig. 12A); spermathecae moderately swollen (Fig. 12B); copulatory ducts basally with a laterally projected bulbus (Fig. 12B); fertilisation ducts emerging at the base of copulatory duct (Fig. 12B).

###### Distribution.

This species is known only from Vale da Castanheira, a 1 km^2^ valley in the north end of Deserta Grande (Fig. 14).

###### Ecology.

Vale da Castanheira is a semi-arid grassland area.

###### Conservation status.

*Hognaingens* was declared Critically Endangered in previous works (Cardoso 2014; Crespo et al. 2014b). Its restricted habitat has been subject to biological invasions since humans set foot in Deserta Grande, with the introduction of herbivore vertebrates and, more recently, of the herb *Phalarisaquatica* L., which grows abundantly throughout the valley, limiting the access of *H.ingens* to shelter below rocks and fissures and displacing native flora. A recovery program of the valley’s vegetation is being conducted, and recent data indicates the spider population is increasing. An ex-situ breeding program is currently being conducted by the Bristol Zoo to safekeep populational levels.

##### ﻿ Hogna insularum

Taxon classificationAnimaliaAraneaeLycosidae

(Kulczynski, 1899)

22F53709-9C4C-5ECA-B94F-40A21CF3195E

**Figure 15.**
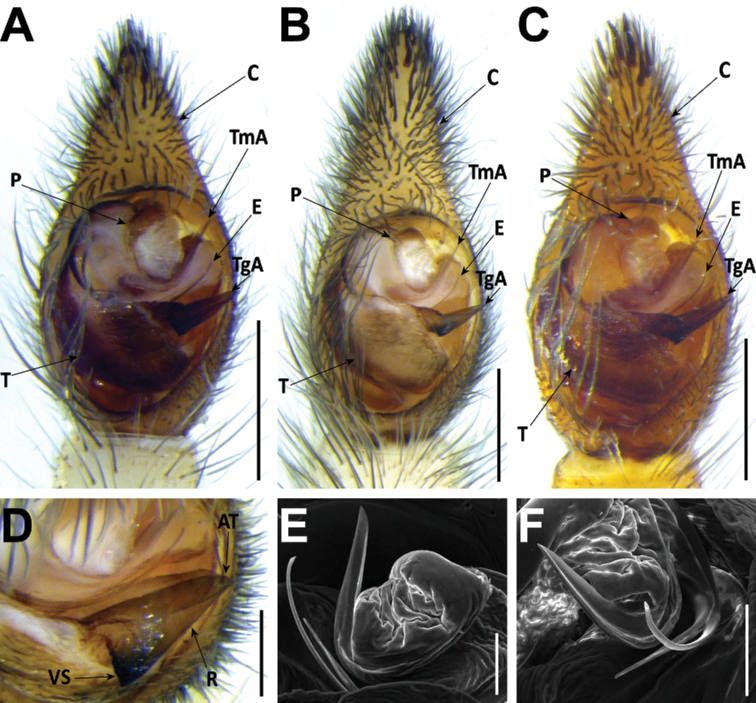
*Hognainsularum*, male pedipalps **A** male from Porto Santo (CRBALC0310), left pedipalp, ventral **B** male from Deserta Grande (CRBALC0305), left pedipalp, ventral **C** male from Bugio (CRBALC0015), left pedipalp, ventral **D** detail of the median apophysis of male from Deserta Grande (CRBALC0305), anteroventral **E**SEM image, right male pedipalp, male from Porto Santo (CRBALC0310), ventral **F** idem, retroventral. Abbreviations, male pedipalp: AT – anterior point, C – cymbium, E – embolus, P – palea, R – ridge, T – tegulum, TA – terminal apophysis, TgA – tegular apophysis, VS – ventral spur. Scale bars: 0.5 mm (**A, B, C**); 0.2 mm (**D**).

**Figure 16.**
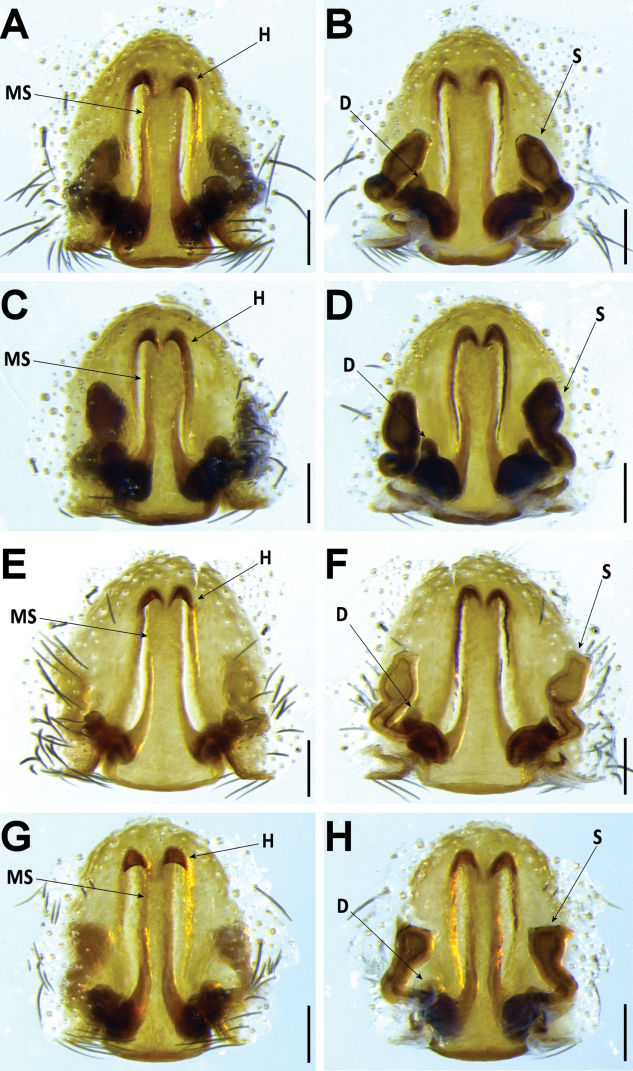
*Hognainsularum*, female genitalia **A, B** female from Bugio (CRBALC0301): **A** epigynum, ventral **B** vulva, dorsal **C, D** female from Deserta Grande (CRBALC0308): **C** epigynum, ventral **D** vulva, dorsal **E, F** female from Madeira (CRBALC0597): **E** epigynum, ventral **F** vulva, dorsal **G, H** female from Porto Santo (CRBALC0300): **G** epigynum, ventral **H** vulva, dorsal. Abbreviations, female genitalia: D – diverticulum, H – epigynal hoods, MS – median septum, S – spermatheca. Scale bars: 0.2 mm.

**Figure 17.**
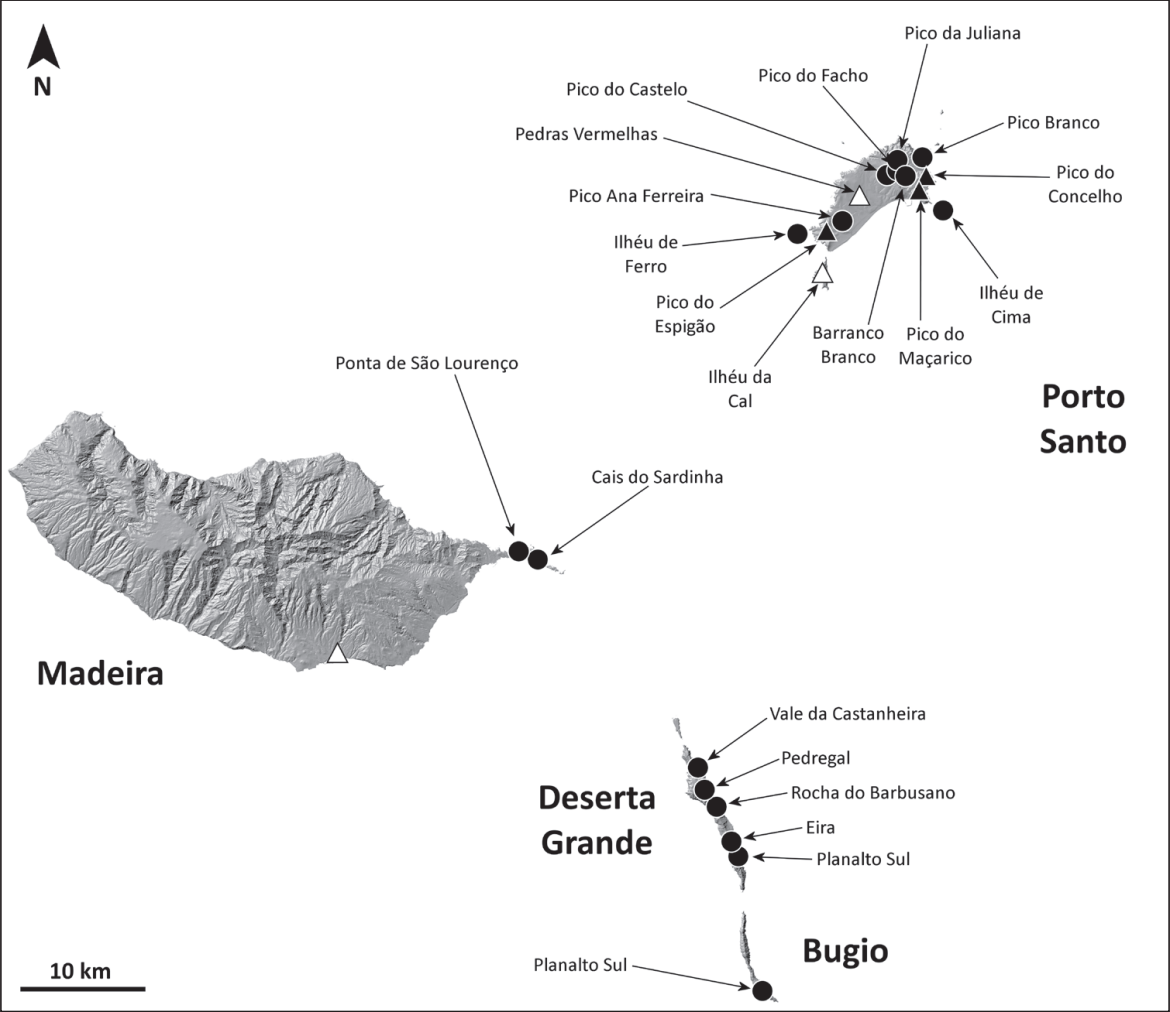
Distribution of *H.insularum*. Circles: present records; black triangles: revised records from literature; white triangles: unconfirmed records from literature.

Figures 15 , 16 , 17

Trochosa insularum Kulczynski, 1899: 429, pl. 9, f. 122, 126 (Dmf).
Hogna insularum Roewer, 1959: 517, fig. 291c, d.
Hogna biscoitoi Wunderlich, 1992: 457, figs 708–709. Syn. nov.
Hogna insularum Wunderlich, 1995: 415, fig. 27 (m).

###### Types.

*Hognabiscoitoi* Holotype ♂ without exact locality, Porto Santo; leg. Winkelmayer, stored at MMF, collection number 24551. Not examined.

***Syntypes***: Madeira • 7 ♀♀ (MIZ217320–217326). Porto Santo • 2 ♂♂ and 14 ♀♀ and 1 juvenile (MIZ217327–217343), leg. Kulczynski, stored at MIZ, collection numbers indicated above. Examined 2 ♂♂ from Porto Santo, 1 ♀ from Madeira.

###### Material examined.

Bugio • Planalto Sul, 32.41228°N, 16.47466°W, 1 ♂ (CRBALC0015) and 1 ♀ (CRBALC0017), 28.VI.2012, hand collecting, leg. I. Silva, 1 ♂ (CRBALC0316: LC229), 1 ♀ (CRBALC0301: LC190) and 2 juveniles (CRBALC0315: LC228, CRBALC0318: LC231), 13.IV.2017, hand collecting, leg. L. Crespo. Deserta Grande • Eira, 32.50993°N, 16.50240°W, 2 juveniles (CRBALC0312: LC282), CRBALC0319: LC232), 11.IV.2017, 1 ♀ (FMNHhttp://id.luomus.fi/HLA.148894), 17.IV.2011, hand collecting, leg. I. Silva; North end, 1 ♂ (MMUE G7508.51), 12.VIII.1981, under stone, leg. J. Murphy; Pedregal (E), 32.54613°N, 16.5234°W, 1 ♀ (CRBALC0308: LC197) and 1 juvenile (CRBALC0306: LC195), 8.IV.2017, hand collecting, leg. L. Crespo & I. Silva, 1 juvenile (CRBALC0285: LC185), 9.IV.2017, hand collecting, leg. L. Crespo; Planalto Sul, 32.50596°N, 16.49986°W, 1 juvenile (CRBALC0413: LC284), 11.IV.2017, hand collecting, leg. L. Crespo & I. Silva; Rocha do Barbusano (S), 32.53168°N, 16.51471°W, 1 juvenile (CRBALC0262: LC175), 10.IV.2017, hand collecting, leg. L. Crespo & I. Silva; Vale da Castanheira, 1 ♂ (FMNHhttp://id.luomus.fi/HLA.148961), 23.IV.2011, hand collecting, leg. I. Silva *et al.*, 1 ♂ (FMNHhttp://id.luomus.fi/HLA.148976), 5.V.2011, pitfall trapping, leg. I. Silva *et al.*, 1 ♀ (FMNHhttp://id.luomus.fi/HLA.148982), 2 ♀♀ (FMNHhttp://id.luomus.fi/HLA.148986), 22.IV.2011, hand collecting, leg. I. Silva; Vale da Castanheira (E), 32.5571°N, 16.52963°W, 1 ♂ (CRBALC0305: LC194), 9.IV.2017, hand collecting, leg. I. Silva; Vale da Castanheira (SE), 32.55078°N, 16.52541°W, 2 ♂♂ (CRBALC0313: LC226, CRBALC0349: LC241) and 1 ♀ (CRBALC0348: LC240), 9.IV.2017, hand collecting, leg. I. Silva. Ilhéu da Cal • 1 ♀ (SMF65693), leg. K. Groh. Ilhéu de Cima • top plateau, 33.05556°N, 16.28097°W, 1 ♀ (CRBALC0019), 9.IV.2012, hand collecting, leg. I. Silva, 1 ♂ (CRBALC0018), 22.V.2011, hand collecting, leg. I. Silva, 1 ♀ (CRBALC0302: LC191) and 4 juveniles (CRBALC0284: LC183, CRBALC0311: LC225, CRBALC0320: LC233, CRBALC0321: LC234), 19.IV.2017, hand collecting, leg. L. Crespo & I. Silva. Ilhéu de Ferro • South tip, 33.03698°N, 16.40814°W, 1 ♀ (CRBALC0317: LC320) and 2 juveniles (CRBALC0265: LC178, CRBALC0266: LC179), 18.IV.2017, hand collecting, leg. L. Crespo & I. Silva. Ilhéu Do Desembarcadouro • 2 ♀♀ (MMUE G7508.50), 28.VIII.1981, under stone, leg. J. Murphy. Madeira • Cais do Sardinha, 32.7419°N, 16.68317°W, 5 juveniles (CRBALC0504: LC242, CRBALC0505: LC243, CRBALC0506: LC244, CRBALC0507: LC245, CRBALC0508: LC246), 30.III.2017, hand collecting, leg. I. Silva; Caniçal, 1 ♀ (MMUE G7572.859), 24.IV.1973, leg. J. Murphy; Caniço, 1 ♀ (MMUE G7508.58), 11.VIII.1981, under stone, leg. J. Murphy; Ponta de São Lourenço, 1 ♂ (MMUE G7508.54), 29.VII.1981, 1 ♀ (MMUE G7508.57), 1.VIII.1981, under stone, leg. J. Murphy, 4 ♂♂ and 5 ♀♀ (FMNHhttp://id.luomus.fi/HLA.156001), 15.V.2011, pitfall trapping, leg. L. Crespo et al., 1 ♂ and 4 ♀♀ (FMNHhttp://id.luomus.fi/HLA.156012), 2.V.2011, hand collecting, leg. L. Crespo et al., 2 ♀♀ (FMNHhttp://id.luomus.fi/HLA.156034), 26.IX.2009, hand collecting, leg. L. Crespo, 1 ♀ (CRBALC0597) and 3 juveniles (CRBALC0599, CRBALC0600, CRBALC0651), 32.749965°N, 16.692817°W, 2.IV.2018, hand collecting, leg. L. Crespo; Ponta do Rosto, 1 ♀ (CRBALC0513: LC251) and 3 juveniles (CRBALC0509: LC247, CRBALC0510: LC248, CRBALC512: LC250), 32.75022°N, 16.70833°W, 30.III.2017, hand collecting, leg. I. Silva. Porto Santo • Rocha de Nossa Senhora, 33.07353°N, 16.3212°W, 1 ♂ (CRBALC0290: LC187) and 1 juvenile (CRBALC0291: LC188), 21.IV.2017, hand collecting, leg. L. Crespo & I. Silva; Pedras Vermelhas, 2 ♂♂ and 1 juvenile (SMF65689), 7.VII.1983, leg. K. Groh; Pico Ana Ferreira, 33.04728°N, 16.37171°W, 1 ♂ (CRBALC0310: LC224), 1 ♀ (CRBALC0327: LC239) and 5 juveniles (CRBALC0303: LC192, CRBALC0307: LC196, CRBALC0326: LC238, CRBALC0309: LC281, CRBALC0430: LC285), 20.IV.2017, hand collecting, leg. L. Crespo & I. Silva; Pico Branco, 33.09428°N, 16.30137°W, 1 ♂ (CRBALC0304: LC193), 21.IV.2017, hand collecting, leg. L. Crespo & I. Silva, 1 ♂ (CRBALC0314: LC227), 23.IV.2017, hand collecting, leg. L. Crespo; Pico da Juliana, 33.09270°N, 16.32186°W, 1 juvenile (CRBALC0286: LC186), 24.IV.2017, hand collecting, leg. L. Crespo; Pico do Castelo, 33.08196°N, 16.33277°W, 2 ♀♀ (CRBALC0300: LC189, CRBALC0322: LC235) and 2 juveniles (CRBALC0267: LC180, CRBALC0268: 181), 17.IV.2017, hand collecting, leg. L. Crespo & I. Silva, 1 ♂ (CRBALC0692), 8.IV.2018, hand collecting, leg. L. Crespo & A. Bellvert; Pico do Concelho, 1 ♀ (SMF65695), 29.VI.1983, leg. K. Groh; Pico do Espigão, 1 ♀ (SMF65692), 1.VII.1983, leg. K. Groh; Pico do Facho, 1 ♀ (SMF65694), 28.VI.1983, leg. K. Groh; Pico do Maçarico [the label reads “Pico dos Magaricos”, therefore we find it necessary to present the correct locality name], 1 ♀ (SMF65691), 10.VII.1983, leg. K. Groh; Terra-Chã (Pico Branco), 33.09447°N, 16.29839°W, 1 ♂ (CRBALC0323: LC236) and 2 juveniles (CRBALC0324: LC327, CRBALC0396: LC283), 21.IV.2017, hand collecting, leg. L. Crespo & I. Silva, 4 juveniles (CRBALC0627, CRBALC0628, CRBALC0630, CRBALC0700), 10.IV.2018, hand collecting, leg. L. Crespo & A. Bellvert. Unknown location • 1 ♀ (NHRS-JUST-000001115), 1 ♂ (MMUE G7508.48), 28.VIII.1981, under stone, leg. J. Murphy, 1 ♂ 1 ♀ and 2 juveniles (SMF34577), 1983, leg. G. Schmidt, 1 ♂ and 1 ♀ (SMF65690), hand collecting, leg. I. Silva, 1 ♀ (NHM 1892.7.9.12.17), leg. W.R.O. Grant.

###### Diagnosis.

*Hognainsularum* can be diagnosed from all other Madeiran *Hogna* by a combination of the following characters: the small to medium size (prosoma length < 10 mm), the aspect of its legs, brown, with black patches (Fig. 27C), male’s embolus thin, with smoothly curved tip (Fig. 15), and female epigyne median septum roughly half as wide (at posterior transverse part) as long (Fig. 16A, C, E, G). It is most similar to *H.heeri* and *H.isambertoi* sp. nov., from which it cannot be somatically differentiated.

###### Redescription.

**Male (CRBALC0310)**: (Fig. 15A, E, F). Total length: 7.8; carapace: 4.6 long, 3.3 wide.

***Colour***: carapace greyish brown, covered with short black setae, with a median cream longitudinal band, anteriorly broadened, covered with short white setae, with suffused greyish brown patches; two yellow marginal bands, with roughly round grey patches, covered with short white setae; four black striae well visible on each flank. Chelicerae brownish orange, with blackish patches, covered in black and white setae. Gnathocoxae greyish yellow, labium overall blackish, with anterior margin greyish yellow; sternum yellow, with a V-shaped grey patch and suffused patches at lateral borders. Legs pale yellow to orange from femora to tibia, with irregular grey suffused patches, metatarsi and tarsi brown. Pedipalps pale yellow except tarsus, brown. Abdomen with a pair of anterolateral black patches, extending laterally into grey flanks, mottled with yellowish patches covered with white setae; a median dark lanceolate patch is bordered by two yellowish longitudinal bands interconnected in anterior half, posteriorly by means of dark chevrons; venter yellowish, with a median dark grey longitudinal band, bordered by small yellowish and grey patches.

***Eyes***: MOQ: MW = 0.8 PW, MW = 1.1 LMP, MW = 1.2 AW; Cl = 0.3 DAME. Anterior eye row slightly procurved.

***Legs***: Measurements: Leg I: 13.6, TiI: 3.1; Leg IV: 14.9, TiIV: 3.1; TiIL/D: 5.5. Spination of Leg I: FeI: d1.1.0, p0.0.1–2; TiI: p1s.0.1s, r1s.0.1s, v2l.2l.2s; MtI: p0.1.1, r0.0.1, v2l.2l.1s. MtI with sparse scopulae in basal half and dense scopulae on distal half.

***Pedipalp***: cymbium with five dark, stout, macrosetae at tip, Fe with two dorsal and an apical row of four spines, Pa with one prolateral spine, Ti with one dorsal and one prolateral spines. Tegular apophysis with ventral spur short, blunt, with a straight ridge leading to a wide apical point (Fig. 15D); terminal apophysis blade-shaped with sharp end (Fig. 15A–C); embolus long and thin, with tip smoothly curved anteriorly (Fig. 15A–C); palea large (Fig. 15A–C).

**Female** (*CRBALC0308*): (Fig. 16C–D). Total length 12.8; carapace: 5.4 long, 4.1 wide.

***Colour***: overall as in male, but darker in legs, chelicera and prosoma. Sternum with a faint V-shaped grey patch. Abdomen is lighter, with central chevrons and ventral longitudinal dark band faded.

***Eyes***: MOQ: MW = 0.8 PW, MW = 1.2 LMP, MW = 1.2 AW; Cl = 0.6 DAME. Anterior eye row slightly procurved.

***Legs***: Measurements: Leg I: 13.8, TiI: 3.1; Leg IV: 16.0, TiIV: 3.3; TiIL/D: 3.7. Spination of Leg I: FeI: d1.1.0, p0.0.2; TiI: p0.1s.0, v2l.2l.2s; MtI: p0.0.1, r0.0.1, v2l.2l.1s. MtI with sparse scopulae in basal half and dense scopulae on distal half.

***Epigyne***: anterior pockets almost touching, short, with lateral borders parallel (Fig. 16C); anterior pocket cavities shallow; median septum with narrow posterior transverse part (Fig. 16C); spermathecae oval or piriform (Fig. 16D); copulatory ducts with small, stout diverticulum ventrally (Fig. 16D); fertilisation ducts emerging at the base of copulatory duct (Fig. 16D).

###### Intraspecific variation.

Carapace length, males: 4.6–6.4, females: 5.1–7.4. Length of cymbium tip of male pedipalp can vary from shorter to longer than the bulbus. In the single available adult female from Madeira, the anterior pockets of the epigyne show slightly divergent lateral borders (posteriorly) (Fig. 16E), while specimens from the remaining islands show parallel lateral borders.

###### Distribution.

This species is known from many locations on all islands of the archipelago except Madeira island, where it is only present at the southeast coastal region (Fig. 17).

###### Ecology.

*Hognainsularum* occurs in a wide variety of habitats, from grasslands, *Erica* shrubland, to secondary forests (in the latter two cases, only in Porto Santo).

###### Conservation status.

*Hognainsularum* was assessed according to the IUCN Red List criteria, with the status of Least Concern (Cardoso et al. 2018c).

###### Comments.

*Hognainsularum* displays remarkable intraspecific variation. In males, both the length of the cymbium tip and the position of the terminal apophysis relative to the embolus are variable (Fig. 15). In females, the epigyne usually presents anterior pockets with anteriorly parallel lateral borders, but a specimen from Madeira shows a posteriorly divergent lateral border. At the same time, the shape of spermathecae seem to vary from ovoid (Fig. 16A–D), to piriform (Fig. 16E, F), to rounded (Fig. 16G, H). Wunderlich (1992) described *H.biscoitoi* based on specimens from Porto Santo. To differentiate it from *H.insularum* he stated that in males “the sickle-shaped apophysis points more to the tip of the cymbium” while in the former species the same structure “(…) is directed more retrolaterally”. For females, although a diagnostic description was provided, the identification key directed to the same image when referring to the epigyne of both *H.insularum* and *H.biscoitoi*. We collected an array of specimens from different localities in Porto Santo (from Pico Ana Ferreira to Pico Branco) and surrounding islets. We did observe male pedipalps with different degrees of inclination of the terminal apophysis and with cymbium tip shorter than the length of the copulatory bulbus (Fig. 15A, C), but both characters were unlinked. We suspect that these traits may be affected by the time from the last moult (e.g., Fig. 15B was most likely a recently moulted individual, given its overall pale coloration). Furthermore, fixation in ethanol might sometimes cause a displacement of sclerites, even if small. Molecular data does not seem to provide any additional evidence regarding the possibility that the specimens from Porto Santo may belong to a different species that those reported form other islands. Unfortunately, we could not examine the type material of *H.biscoitoi* stored at the Funchal Municipal Museum, since it does not loan type material for study. Based on the variability in the supposedly diagnostic features and the lack of genetic divergence, we consider *H.biscoitoi* as a junior synonym of *H.insularum*.

##### ﻿ Hogna isambertoi

Taxon classificationAnimaliaAraneaeLycosidae

Crespo sp. nov.

FF3689AA-17FA-56DB-BC68-1B299258C429

http://zoobank.org/87BB2C30-D40D-4B5D-92F5-D7D23ED9A7BC

**Figure 18.**
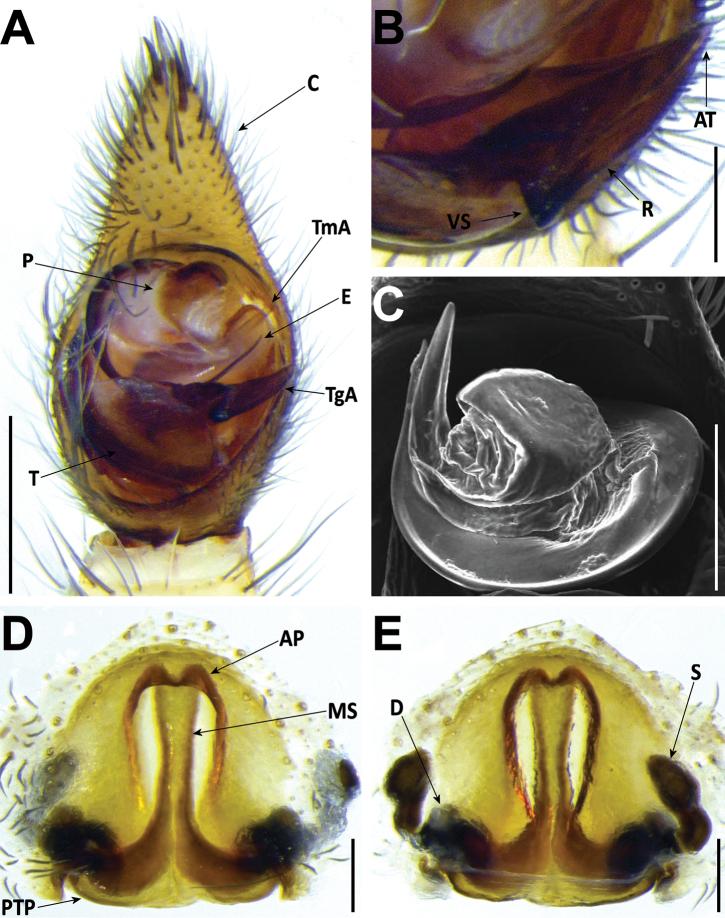
*Hognaisambertoi* sp. nov. **A–C** male (SMF): **A** left male palp, ventral **B** detail of the median apophysis, anteroventral **C**SEM image, right male palp, ventral **D, E** female (SMF): **D** epigynum, ventral **E** vulva, dorsal. Abbreviations, male palp: C – cymbium, E – embolus, MA – median apophysis, P – palea, T – tegulum, TA – terminal apophysis. Abbreviations, female genitalia: D – diverticulum, H – epigynal hoods, MS – median septum, S – spermatheca. Scale bars: 0.5 mm (**A**); 0.2 mm (**B–E**).

**Figure 19.**
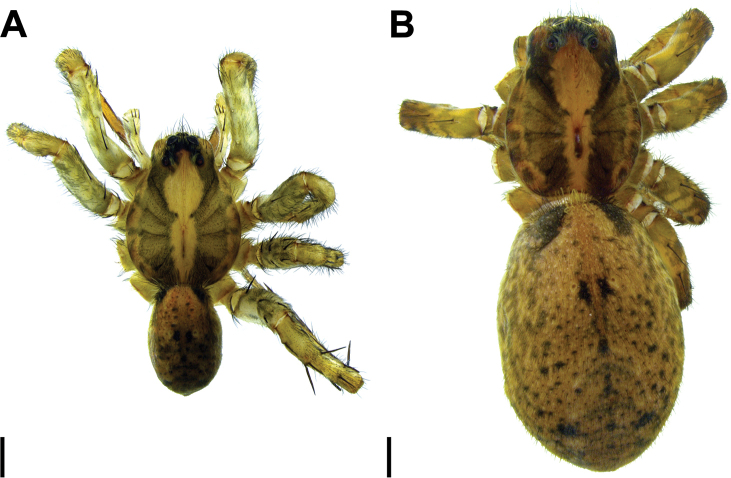
*Hognaisambertoi* sp. nov. **A** male habitus, dorsal (SMF) **B** female habitus, dorsal (SMF). Scale bars: 1 mm.

**Figure 20.**
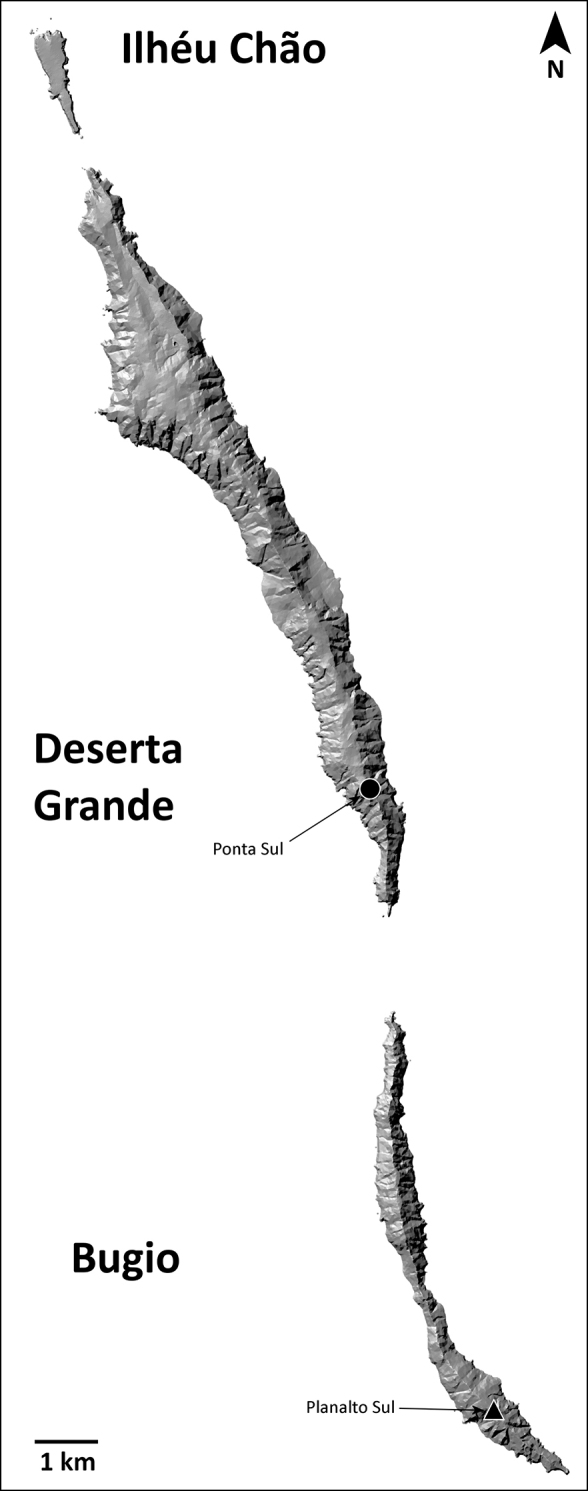
Distribution of *H.isambertoi* sp. nov. Circles: present records; black triangle: revised record from literature.

Figures 18 , 19 , 20

Hogna heeri Crespo et al. 2013: 18 (m, misidentification).

###### Types.

***Holotype***: Deserta Grande • 1 ♂, Ponta Sul, 32.49562°N, 16.49562°W, coll. 4.XI.2017, leg. I. Silva, stored at SMF, collection number to be set after publication. ***Paratypes***: Bugio • 1 ♂ (SMF), Planalto Sul, 3.XII.2012, hand collecting, leg. I. Silva. Deserta Grande • Planalto Sul, 1 ♀ (SMF), 12.XI.2017, hand collecting, leg. I. Silva.

###### Material examined.

Deserta Grande • Planalto Sul, 1 juvenile (CRBALC0610: LC330), 12.XI.2017, hand collecting, leg. I. Silva.

###### Diagnosis.

*Hognaisambertoi* sp. nov. can be distinguished from all other Madeiran *Hogna* by its genitalia. In males, the embolus is thick and tilted anteriorly at the tip and a tegular apophysis with a very short ventral spur (Fig. 18A, C). In females, the epigynal anterior pockets show convergent lateral borders and the median septum has a wide posterior transverse part (Fig. 18D). It is most similar to *H.heeri* and *H.insularum*, from which it cannot be somatically differentiated.

###### Description.

**Male holotype**: (Figs 18A–C, 19A). Total length: 7.4; carapace: 4.6 long, 3.2 wide.

***Colour***: carapace greyish brown, covered with short black setae, with a median yellow longitudinal band, anteriorly broadened, covered with short white setae; two yellow marginal bands, suffused with grey patches, covered with short white setae; four black striae well visible on each flank. Chelicerae yellow, with grey suffused patches, covered in black and white setae. Gnathocoxae and labium overall pale yellow, with posterior margin with suffused grey patch; sternum pale yellow, with V-shaped grey patch. Legs pale yellow, with irregular grey suffused patches, except anterior metatarsi and tarsi, yellowish orange. Pedipalps yellow. Abdomen with a pair of anterolateral black patches, extending laterally into grey to black flanks; a median faint dark lanceolate patch is bordered by two yellowish longitudinal bands interconnected in anterior half, posteriorly by means of dark chevrons; venter yellowish, with large blackish patches near spinnerets and small patches medially.

***Eyes***: MOQ: MW = 0.8 PW, MW = 1.1 LMP, MW = 1.1 AW; Cl = 0.5 DAME. Anterior eye row slightly procurved.

***Legs***: Measurements: Leg I: 11.7, TiI: 2.6; Leg IV: 13.8, TiIV: 2.8; TiIL/D: 6.6. Spination of Leg I: FeI: d1.1.1, p0.0.1; TiI: p1.0.1, v2l.2l.2s; MtI: p0.0.1, r0.0.1, v2l.2l.1s. MtI with sparse scopulae in basal half and dense scopulae on distal half.

***Pedipalp***: cymbium with one spine along prolateral rim and five dark, stout, macrosetae at tip, Fe with two dorsal and an apical row of four spines. Tegular apophysis with ventral spur very short, blunt, with a concave ridge leading to a thin apical point (Fig. 18A, B); terminal apophysis in close apposition with subterminal apophysis, which is blade-shaped with blunt end (Fig. 18A, C); embolus long and thick, with tip tilted anteriorly (Fig. 18A); palea large (Fig. 18A).

**Female paratype**: (Figs 18D, E, 19B). Total length 12.1; carapace: 4.7 long, 3.6 wide.

***Colour***: overall as in male, but darker in legs, chelicera and prosoma, where additional faint striae are present. Abdomen is lighter, with central chevrons faded, possibly due to pregnancy and correspondent tegument extension.

***Eyes***: MOQ: MW = 0.8 PW, MW = 1.2 LMP, MW = 1.8 AW; Cl = 0.4 DAME. Anterior eye row slightly procurved.

***Legs***: Measurements: Leg I: 9.9, TiI: 1.7; Leg IV: 13.0, TiIV: 2.6; TiIL/D: 3.2. Spination of Leg I: FeI: d1.1.0, p0.0.1–2; TiI: p0.0.0–1, v2l.2l.2s; MtI: p0.0.1, r0.0.1, v2l.2l.1s. MtI with sparse scopulae in basal half and dense scopulae on distal half.

***Epigyne***: anterior pockets touching, short, with lateral borders parallel (Fig. 18D); anterior pocket cavities deep; median septum with wide posterior transverse part (Fig. 18D); spermathecae oval (Fig. 18E); copulatory ducts simple (Fig. 18E); fertilisation ducts emerging at the base of copulatory duct (Fig. 18E).

###### Etymology.

the specific epithet is a patronym in honour of Isamberto Silva, who not only collected the only known specimens of this species, but has provided invaluable support during field work.

###### Intraspecific variation.

Carapace length, males: 4.1–4.3.

###### Distribution.

This species is known only from the southernmost part of Deserta Grande and Bugio (Fig. 20).

###### Ecology.

*Hognaisambertoi* sp. nov. occurs in arid, coastal scarps, with reduced vegetation cover.

###### Conservation status.

the species seems to be restricted to a very small area, equivalent to an Extent of Occurrence and Area of Occupancy of 8 km^2^ in two locations, both threatened by the effects of increasing aridification. The trends are unknown, but it is uncertain if the scarcity of specimens is due to rarity, or the fact that it seems to be a late autumn / early winter species, when collecting effort has been low. If the decline is confirmed the status might be Endangered, if not it might be Near Threatened.

##### ﻿ Hogna maderiana

Taxon classificationAnimaliaAraneaeLycosidae

(Walckenaer, 1837)

42E4561E-DAC1-54CF-8A4A-4BF65EA2FA6B

**Figure 21.**
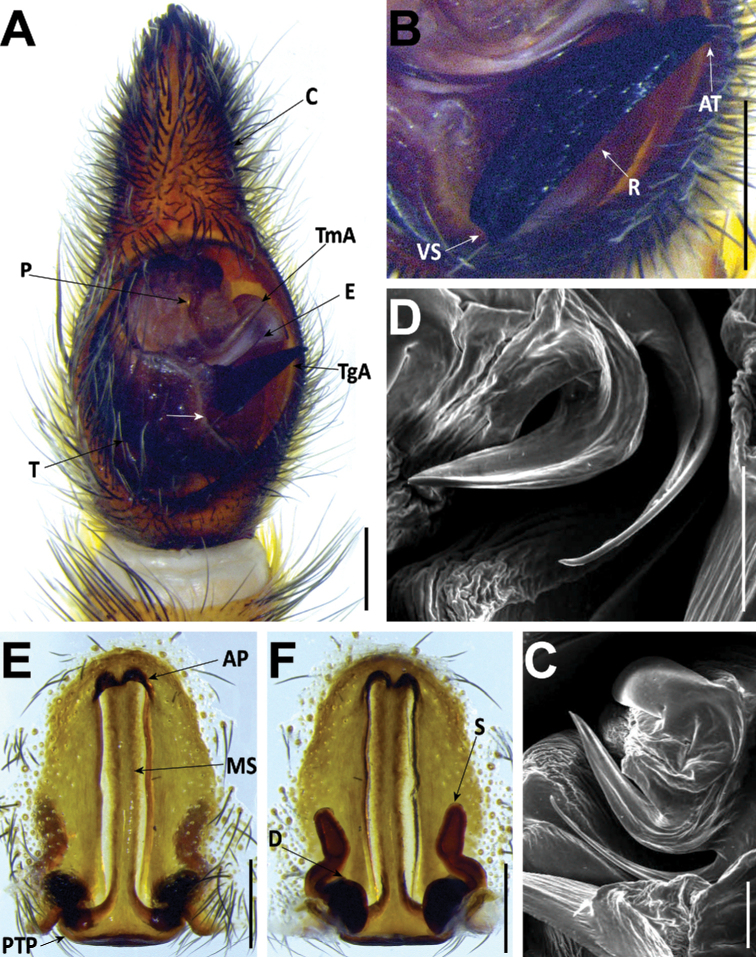
*Hognamaderiana***A–D** male (CRBALC0734): **A** left male palp, ventral (white arrow points to a tegular concavity that may be helpful for diagnosis) **B** detail of the median apophysis, anteroventral **C**SEM image, right male palp, ventral **D** idem, retroventral **E, F** female (CRBALC0717): **E** epigynum, ventral **F** vulva, dorsal. Abbreviations, male pedipalp: AT – apical point, C – cymbium, E – embolus, P – palea, R – ridge, T – tegulum, TA – terminal apophysis, TgA – tegular apophysis, VS – ventral spur. Abbreviations, female genitalia: D – diverticulum, H – epigynal hoods, MS – median septum, S – spermatheca. Scale bars: 0.5 mm (**A, B, E, F**); 0.2 mm (**C, D**).

**Figure 22.**
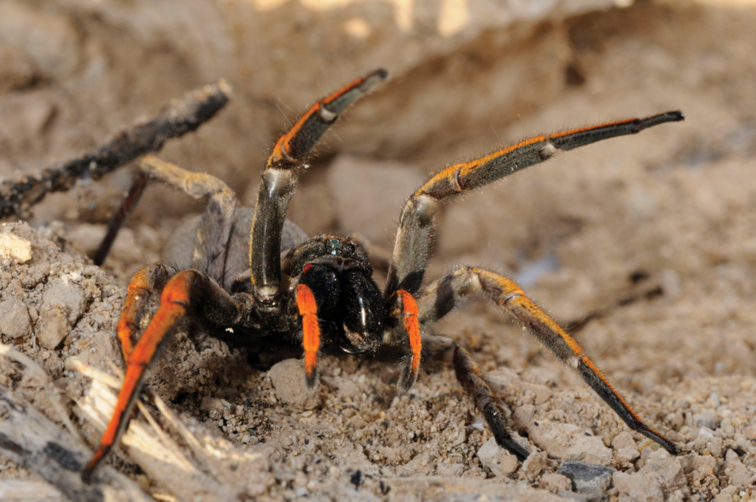
Photograph of *H.maderiana*. Female specimen in the field. Photograph credit Pedro Cardoso.

**Figure 23.**
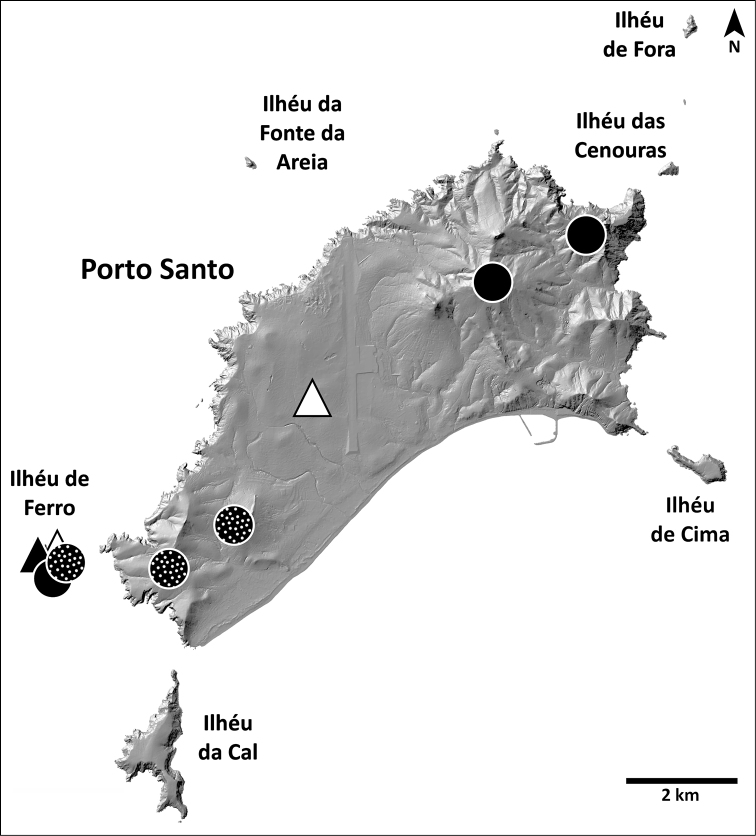
Distribution of *H.maderiana*. Black circles: present records; dotted circles: records from only leg samples; black triangles: revised records from literature; white triangles: unconfirmed records from literature.

Figures 21 , 22 , 23

Lycosa tarentuloides maderiana Walckenaer, 1837: 291 (Df).
Lycosa tarentuloides maderiana Blackwall, 1857a: 282 (Dm).
Tarentula maderiana Simon, 1864: 350.
Lycosa maderiana Simon, 1898: 346.
Trochosa maderiana Kulczynski, 1899: 426, pl. 9, fig. 119–120 (mf).
Isohogna maderiana Roewer, 1955: 241.
Isohogna maderiana Roewer, 1960: 569, fig. 319a–c (mf).
Hogna schmitzi Wunderlich, 1992: 462, fig. 721–723 (Dmf). Holotype ♂ examined, Porto Santo, 8–11.VII.1983; leg. K. Groh, stored at SMF, collection number 37639. New synonymy.

###### Types.

***Holotype***: Not examined, supposed lost.

###### Material examined.

Ilhéu de Ferro • 1 ♂ and 1 ♀ (SMF37637), 3.VII.1983, leg. K. Groh., 1 ♂ (CRBALC0013), 33.03698°N, 16.40814°W, 6.IV.2011, hand collecting, leg. I. Silva. Porto Santo • Pico Branco, 33.09366°N, 16.30776°W, 1 ♂ (CRBALC0734) and 2 ♀♀ (CRBALC0704, CRBALC0717), 10.IV.2018, hand collecting, leg. L. Crespo & A. Bellvert; Pico do Facho, 1 ♂ (SMF63869), 31.X.1972; (unknown location), 1 ♀ (MNHNP AR16184), 27.III.1959, leg. A. Vandel, 2 ♀♀ and 2 juveniles (FMNHhttp://id.luomus.fi/KN.23945), 4.X.1959, 1 ♀ (SMF34482), VII.1983, 1 ♀ (SMF36760), 26.X.1985, leg. G. Schmidt, 1 ♀ (SMF37636) and 2 juveniles (SMF37638), leg. K. Groh, 8 ♂♂ and 11 ♀♀ (NHM, in ethanol), VI.1962, leg. S.W. Bristowe, 1 ♀ (NHM), VII.1963, leg. B.M. Cliffton, 2 ♂♂ and 2 ♀♀ (NHM 1892.7.9.12.17), leg. W.R.O. Grant, 1 ♀ (NHM), 12.VI.1964, 1 ♂ and 1 ♀ (NHM, mounted dry).

###### Diagnosis.

*Hognamaderiana* can be distinguished from all other Madeiran *Hogna* by a combination of the following characters: the large size (prosoma length > 10 mm), the presence of conspicuous orange setae (Fig. 27A), and its genitalia. In males by a combination of a smoothly curved tip of the embolus, a long, blunt ventral spur, and a deeper tegular concavity (Fig. 21A–D). In females by epigyne with median septum more than twice as long as wide (at posterior transverse part) (Fig. 21E, F).

###### Redescription.

**Male (CRBALC0734)**: (Fig. 21D, E). Total length: 19.5; carapace: 11.9 long, 8.9 wide.

***Colour***: carapace brown, with short black setae covering flanks, short white setae present posteriorly, anteriorly and laterally, long black setae are present anteriorly or scattered around median band; median yellow longitudinal band present but faint, covered with short white setae and scattered long black setae, anteriorly broadened; marginal bands indistinct, made apparent only by the cover of short white setae, long black setae also present laterally; four darker lateral bands visible, but without striae. Chelicerae black, apically dark brown, covered in black and yellow setae. Gnathocoxae very dark orange-brown, labium blackish; sternum brown, medially lighter, but without any stripe. Legs yellow to orange-brown, without annulations, with anterior tibiae, all metatarsi and tarsi dark brown, and covered dorsally with yellow setae (probably orange in living or fresh specimen). Pedipalpal femur, patella, and tibia as legs, cymbium darker, yellow setae present in all segments except femur. Abdomen with a pair of anterolateral black patches, extending laterally into grey flanks; a median yellow lanceolate patch is bordered by few whitish patches; venter greyish, darker near spinnerets.

***Eyes***: MOQ: MW = 0.7 PW, MW = 1.2 LMP, MW = 1.2 AW; Cl = 0.5 DAME. Anterior eye row slightly procurved.

***Legs***: Measurements: Leg I: 36.7, TiI: 8.85; Leg IV: 37.3, TiIV: 8.1; TiIL/D: 4.4. Spination of Leg I: FeI: d1.1.0, p0.0.2; TiI: p1.0.1, r1.0.0, v2s.2s.2s; MtI: p0.0.1, r0.0.1, v2s.2s.1s. MtI with very dense scopulae.

***Pedipalp***: cymbium with one prolateral spine and six dark, stout, macrosetae at tip, Fe with two dorsal and an apical row of four spines, Pa with one prolateral spine, Ti with one dorsoprolateral and one prolateral spines. Tegular apophysis with ventral spur long, blunt, with a straight ridge leading to a wide apical point (Fig. 21A, B); terminal apophysis blade-shaped with sharp end (Fig. 21A–D); embolus long, with tip directed anterolaterally (Fig. 21A–D); palea small (Fig. 21A).

**Female (CRBALC0717)**: (Fig. 21E, F). Total length 23.5; carapace: 11.3 long, 8.3 wide.

***Colour***: overall as in male, with the following differences: median yellow longitudinal band in prosoma clear. Cheliceral setae black. Legs with few faint greyish patches in femora. Abdominal pattern overall greyish, darker near spinnerets, with patches unapparent.

***Eyes***: MOQ: MW = 0.7 PW, MW = 1.1 LMP, MW = 1.2 AW; Cl = 0.4 DAME. Anterior eye row slightly procurved.

***Legs***: Measurements: Leg I: 30.3, TiI: 7.2; Leg IV: 33.9, TiIV: 7.4; TiIL/D: 3.5. Spination of Leg I: FeI: d1.1.0, p0.0.2; TiI: 0.1s.0, v2s.2s.2s; MtI: p0.0.1, r0.0.1, v2l.2s.1s. MtI with very dense scopulae.

***Epigyne***: anterior pockets touching, short, with lateral borders parallel (Fig. 21E); anterior pocket cavities deep; median septum with narrow posterior transverse part (Fig. 21E); spermathecae elongated (Fig. 21F); copulatory ducts with very small diverticulum ventrally (Fig. 21F); fertilisation ducts emerging at the base of copulatory duct (Fig. 21F).

###### Intraspecific variation.

Carapace length, males: 11.9–14.4, females: 11.0–11.5.

###### Distribution.

This species is known from the island of Porto Santo and one of its surrounding islets, Ilhéu de Ferro (Fig. 23).

###### Ecology.

*Hognamaderiana* can be found in open habitats, such as grasslands, shrubland or sand banks. Very common even in relatively disturbed habitats across Porto Santo.

###### Conservation status.

*Hognamaderiana* was assessed according to the IUCN Red List criteria as *H.schmitzi* (Cardoso et al. 2018d), with the status of Least Concern.

###### Comments.

As mentioned above (see remarks on *H.blackwalli*), the large specimens with striking orange coloration in legs from Porto Santo island and its neighbouring islet Ilhéu de Ferro were known to pioneer arachnologists. The original, somewhat obscure, description by Walckenaer described a 2.5 cm spider (“1 pouce”) with reddish brown legs (“Pattes rouges, lavées de brun en dessus (…)”), from the island of Madeira (“Ile de Madère”). After this, Blackwall was the first to provide a clear description of this taxon, while at the same time stating that it was collected in the island of Porto Santo, not Madeira. Subsequent authors reported additional material from either Porto Santo or Ilhéu de Ferro (Johnson 1863; Kulczynski 1899). Wunderlich considered *H.blackwalli* a junior synonymy of *H.maderiana* based on the wrong assumption that previous authors repeatedly misidentified *H.maderiana* from Porto Santo, assigning *H.maderiana* to the large species with annulated legs from Madeira island. Following synonymy, Wunderlich himself named the large species from Porto Santo as *H.schmitzi*. As a matter of fact, the only indication of the presence of a large spider with reddish leg coloration in Madeira island is Walckenaer’s original description. Unfortunately, Walckenaer’s type seems to be lost. However, Simon most likely examined it because he stated that “*L.maderiana* Walck. est, en grande partie, revêtu, en dessus, de pubescence courte d’un beau rouge orange.” (Simon 1898: 332). The two large species are easy to distinguish, the only misidentification between them being made by Thorell, who identified *H.blackwalli* from Madeira as *Trochosamaderiana* (Thorell 1875). We argue that the presence of *H.maderiana* in the island of Madeira reported in Walckenaer’s original description was likely a labelling mistake or a misinterpretation, and probably referred to the archipelago.

##### ﻿ Hogna nonannulata

Taxon classificationAnimaliaAraneaeLycosidae

Wunderlich, 1995

75E821A9-FADB-536C-9947-3316B1CFED95

Figures 24 , 25

###### Types.

***Holotype***: Madeira • 1 ♂, coll. 25–30.IV.1957, leg. Roewer, stored at SMF, collection number 10754. Examined.

###### Material examined.

Madeira • Câmara de Lobos, 32.6525°N, 16.96683°W, 1 ♂ (CRBALC0703: LC326), 27.V.2018, hand collecting, leg. É. Pereira, 1 ♂ (CRBALC0701: LC325, CRBALC0702: LC324), 29.V.2018, hand collecting, leg. I. Silva & É. Pereira, 1 ♂ (CRBALC0608: LC328), 21.VI.2017, hand collecting, leg. I. Silva, 1 ♂ (CRBALC0607: LC327), 11.VIII.2017, hand collecting, leg. I. Silva.

###### Diagnosis.

**Figure 24.**
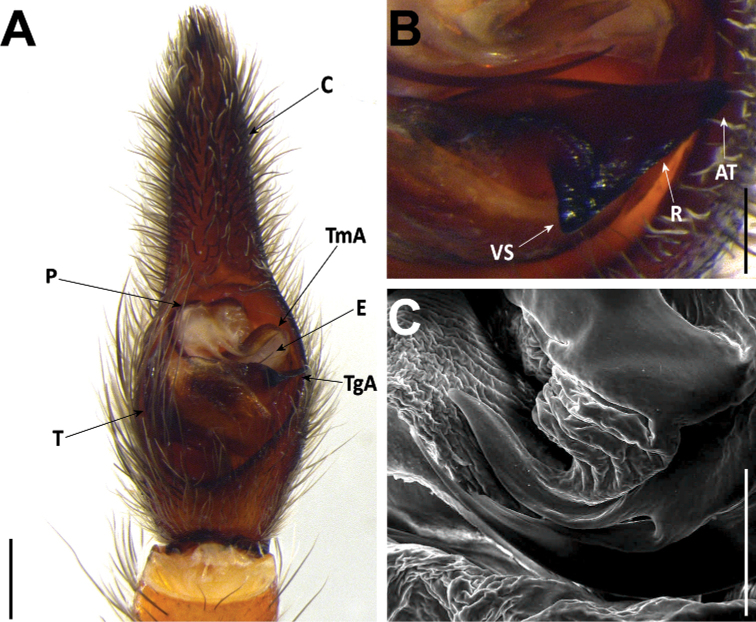
*Hognanonannulata* male (CRBALC0701): **A** left male pedipalp, ventral **B** detail of the median apophysis, anteroventral **C**SEM image, right male pedipalp, ventral. Abbreviations, male pedipalp: AT – apical point, C – cymbium, E – embolus, P – palea, R – ridge, T – tegulum, TA – terminal apophysis, TgA – tegular apophysis, VS – ventral spur. Scale bars: 0.5 mm (**A**); 0.2 mm (**B, C**).

*Hognanonannulata* can be distinguished from all other Madeiran *Hogna* by the aspect of its legs, without annulations or bright yellow or orange setae (Fig. 24D). In addition, males have an elongate cymbium tip, clearly longer than the length of the alveolus of the bulb (Fig. 24A). We could not revise any female materials, for which we propose that the leg aspect can be used to diagnose females.

###### Redescription.

**Male (CRBALC0701)**: (Fig. 24). Total length: 18.6; carapace: 10.3 long, 8.2 wide.

***Colour***: carapace greyish brown with transverse yellowish bands, generally covered with short black setae, except anteriorly and laterally, provided with short white setae and long black setae; median yellow longitudinal band present, anteriorly broadened, with suffused greyish brown patches; two yellow marginal bands, suffused with greyish brown patches; ca. seven faint blackish striae on each flank. Chelicerae blackish to dark brown, mostly covered with black and white setae. Gnathocoxae very dark orange-brown, labium blackish; sternum yellowish grey, with a faint, longitudinal yellow stripe extending to less than half of sternum length. Legs yellow to brown, without any clearly coloured patch, just scattered areas suffused with grey, grey setae present in tibia, metatarsus and tarsus. Pedipalpal femur, patella, and tibia yellow, except cymbium, brown. Abdomen with both short and long black setae, additionally with short greyish white setae; with a pair of anterolateral faint blackish patches, extending laterally into grey flanks, interspersed with greyish white patches; a median greyish lanceolate patch is bordered by two yellowish longitudinal bands interconnected in anterior half, posteriorly by means of faint dark chevrons; venter yellowish except around spinnerets, dark grey, with small blackish patches scattered laterally.

***Eyes***: MOQ: MW = 0.8 PW, MW = 1.2 LMP, MW = 1.3 AW; Cl = 0.7 DAME. Anterior eye row slightly procurved.

***Legs***: Measurements: Leg I: 40.9, TiI: 10.8; Leg IV: 43.0, TiIV: 9.8; TiIL/D: 8.8. Spination of Leg I: FeI: d1.1.0, p0.0.2; TiI: p1.0.1, v2s.2s.2s; MtI: p1.0.1, r1.0.1, v2s.2s.1s. MtI with very dense scopulae.

***Pedipalp***: cymbium with two prolateral spines, one basal, the other at rim, apically with four dark macrosetae, Fe with two dorsal and an apical row of four spines, Pa with one prolateral spine, Ti with one dorsal, one dorsoprolateral, and one prolateral spines. Tegular apophysis with ventral spur short, blunt, with a short straight ridge leading to a wide apical point; terminal apophysis separated from subterminal apophysis due to a clearly visible excavation, blade-shaped with sharp end; embolus moderately elongated, with tip directed anteriorly; palea small.

**Female**: We could not revise any female material.

###### Intraspecific variation.

Carapace length, males: 7.2–11.3. Smaller males have proportionally longer tibial spines than longer males.

###### Distribution.

**Figure 25.**
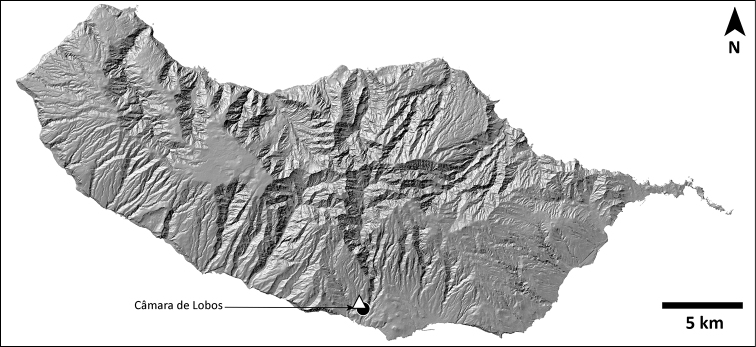
Distribution of *H.nonannulata*. Black circle: present record; white triangle: unconfirmed record from literature.

This species is known from the southern coastal area of Câmara de Lobos in the island of Madeira (Fig. 25).

###### Ecology.

*Hognanonannulata* can be found in coastal shrub- or grassland and rocky areas.

###### Conservation status.

It was not previously possible to assess *H.nonannulata* according to the IUCN Red List criteria given the scarcity of past information, hence a status of Data Deficient was suggested (Cardoso et al. 2018e). Its known distribution is now limited to the area of Camara de Lobos in the southern coast of Madeira Island, an area with no remaining natural habitat beyond the rocky areas. With an EOO and AOO of 4 km^2^ and a single location threatened by urban and agricultural pressure, if the trend of the species is negative its status might be Critically Endangered.

### ﻿Key to the *Hogna* species endemic to the Madeira Archipelago

**Table:** 

1	Species from Porto Santo.	**2**
–	Species from Madeira or Desertas.	**3**
2	Large species (prosoma length > 10 mm), legs furnished with orange setae (Fig. 27A).	***H.maderiana***
–	Small to medium species (prosoma length < 10 mm), legs with whitish setae (Fig. 27C).	***H.insularum***
3	Species from Madeira.	**4**
–	Species from Desertas.	**7**
4	Legs with a small, bright yellow patch of setae at joints of anterior metatarsus and pedipalp (Fig. 26A).	***H.blackwalli***
–	Species without bright yellow patches of setae in anterior legs.	**5**
5	Legs without any reticulated or annulated pattern (Fig. 26D)	***H.nonannulata***
–	Legs with reticulated or annulated pattern.	**6**
6	Male with straight embolus (Wunderlich 1992: 595, fig. 720). Female epigynal anterior pockets with highly divergent lateral borders (Fig. 9A). Species from montane habitats.	***H.heeri***
–	Male with embolus smoothly curved (Fig. 15). Female epigynal anterior pockets with parallel lateral borders (Fig. 16C). Species from southeastern coastal grassland habitats.	***H.insularum***
7	Very large species (prosoma length > 14 mm). Black legs with white patches (Fig. 26C).	***H.ingens***
–	Smaller species (prosoma length < 10 mm).	**8**
8	Male pedipalp with embolus smoothly curved (Fig. 15). Female epigyne with median septum roughly half as wide (at posterior transverse part) as long (Fig. 16A, C, E, G).	***H.insularum***
–	Male pedipalp with embolus straight or with only tilted tip. Female epigyne with median septum almost as wide (at posterior transverse part) as long (Figs 9A, B, 18D, E).	**9**
9	Male pedipalp with embolus with tip tilted anteriorly (Fig. 18A, C). Female epigynal anterior pockets with convergent lateral borders (Fig. 18D)	***H.isambertoi* sp. nov.**
–	Male pedipalp with straight embolus (Wunderlich 1992: 595, fig. 720). Female epigynal anterior pockets with highly divergent lateral borders (Fig. 9A)	***H.heeri***

## ﻿Discussion

### ﻿Origins of Madeiran *Hogna*

Our analyses support the long-standing view that the genus *Hogna* is a paraphyletic assemblage in much need of a thorough taxonomic revision that could establish its limits and diagnosis. Unfortunately, only 18 species of *Hogna* were represented by at least one DNA sequence in public repositories, out of the 228 currently valid species and subspecies, excluding Madeiran ones (World Spider Catalog 2021). Albeit with low support, our results suggested a strong geographic component in the phylogenetic relationship of *Hogna* species, recovering mixed genera clades from the same region (e.g., North America, South America or Australia). Madeiran species were consistently recovered by all analyses as closely related to the type species of the genus, *H.radiata*, represented in the analyses by specimens from the Iberian Peninsula, yet both the monophyly of the Madeiran species and their relation with *H.radiata* are poorly supported. Although our sampling is far from being representative of the *Hogna* diversity in the western palearctic (only two species of 45 described were included), the results are congruent with the Iberian Peninsula as a colonisation source of Madeiran species. This biogeographic connection has been recently confirmed for the endemic Madeiran species of the spider genus *Dysdera*, and was most likely favoured by the predominant aerial and marine currents in the region (Crespo et al. 2021).

**Figure 26.**
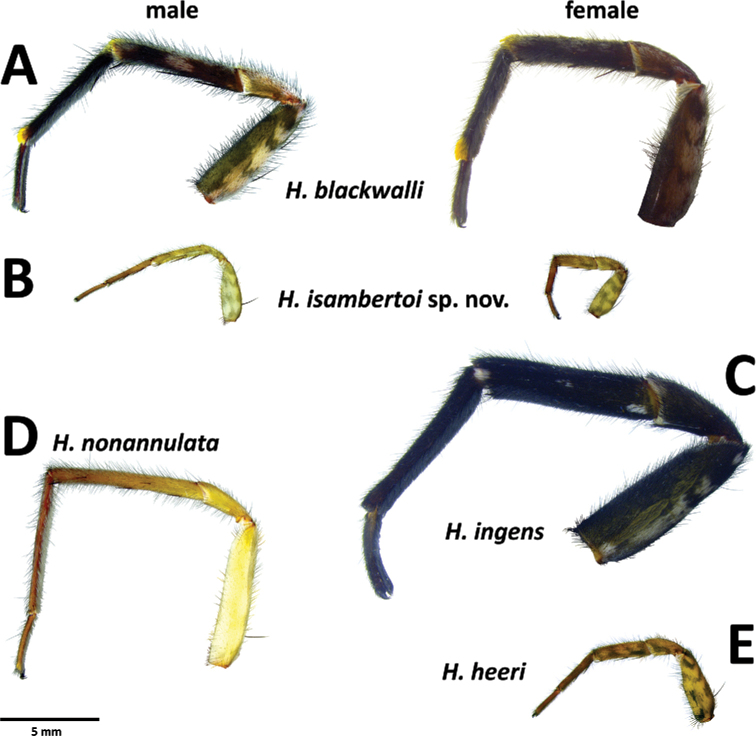
Plate with photographs of the lateral view of the leg I for easily diagnosable species.

Our time estimates suggest a colonisation of the archipelago by the late Miocene (but note the large confidence intervals recovered). Interestingly, this sub-epoch coincides with an episode of major global cooling that brought about dramatic changes in the ecosystems, which included the expansion of grasslands and the associated fauna (see Herbert et al. 2016 and references therein). The increase in the amount of habitat types preferred by many wolf spider species may have facilitated the expansion and diversification of lycosids into the Mediterranean region and eventually the colonisation of the Madeiran Archipelago. In this regard, it is worth mentioning that the origin of the western Mediterranean species of the other genus of large wolf spiders, *Lycosa* Latreille, 1804, seems also to trace back to the late Miocene (Planas et al. 2013).

Model-based analyses recovered the monophyly of all Madeiran endemics, which would suggest a single colonisation event of the archipelago. This result was disputed by parsimony analysis, which suggested at least two different events by placing the Iberian *H.radiata* as sister to the *ingens* clade. None of these alternative arrangements, however, received high support. Conversely, the existence of two well-defined lineages, the *ingens* and *maderiana* clades, were supported in all analyses. Interestingly, our analyses also signalled multiple colonisations of another volcanic archipelago, the Galapagos Islands. Up to seven endemics species are known from this Pacific archipelago, which include species adapted to habitats at different altitudes (Baert et al. 2008). All our analyses supported the independent colonisation of the Galapagos by at least two or even three different ancestors, one of which resulted in local diversification. Multiple island colonisation should not be unexpected in wolf spiders, given their good dispersal ability and frequent use of ballooning by many species (Richter 1970; Greenstone 1982; Bonte and Maelfait 2001; Bonte et al. 2006), although it was never assessed in *Hogna*.

**Figure 27.**
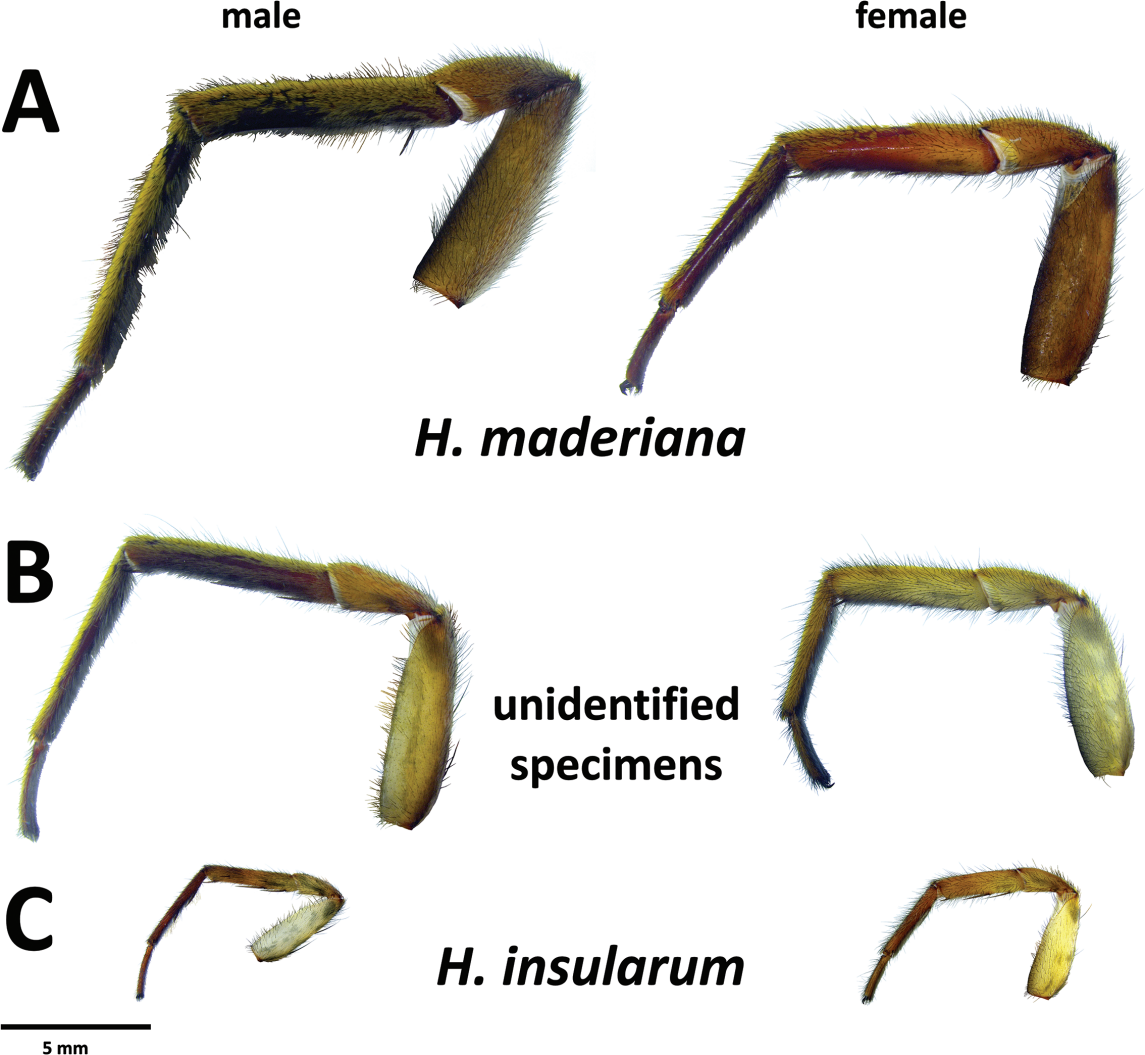
Plate with photographs of the lateral view of the leg I for the complex of *H.maderiana*, *H.insularum* and their intermediate forms.

Regardless of the actual number of colonisations, *Hogna* underwent processes of local diversification, as illustrated by the *ingens* clade. Similarly, to what has been observed in endemic *Hogna* from the Galapagos (De Busschere et al. 2010), Madeiran endemics show a certain ecological differentiation associated to elevation, some species are found in montane habitats (*H.heeri*, *H.blackwalli* sp. rev. and *H.ingens*), while other are mostly found in coastal areas (*H.isambertoi* sp. nov. and *H.nonannulata*). Body size is another functional trait with a noticeable variation across Madeiran *Hogna*, *H.ingens* and *H.maderiana* can be considered giant species for *Hogna* standards (> 10 mm of carapace length), while *H.blackwalli* (7.3–10.4 mm) and *H.nonannulata* (7.2–11.2 mm) are medium-large, and *H.insularum* (4.1–4.7 mm), *H.heeri* (5.2–5.8 mm), and *H.isambertoi* sp. nov. (4.1–4.7 mm) are small. Often sympatric species have disparate sizes, as is the case in Porto Santo with *H.maderiana* and *H.insularum*, or in Deserta Grande with *H.ingens* and *H.insularum*, or even in Madeira with *H.blackwalli*, and *H.heeri*. Yet, it also happens that in Deserta Grande (only in the southern end) two very similar species, *H.insularum* and *H.isambertoi* sp. nov., share the same habitat. And in Bugio island, an even smaller and steeper island than Deserta Grande, the three small species of the archipelago, *H.heeri*, *H.insularum*, and *H.isambertoi* sp. nov., are found together. The few specimens available of *H.isambertoi* sp. nov. and the single specimen of *H.heeri* from Bugio were all collected in late autumn, which, hypothetically, might suggest phenological displacement against the spring-dominant *H.insularum*.

Within the *ingens* clade, the only well-supported sister group relationship is between *H.blackwalli* and *H.nonannulata*, which can represent an example of ecological shift within the same island, from the ancestral open habitat represented by the coastal species *H.nonannulata*, to the laurel forest habitats inhabited by *H.blackwalli* This is a more plausible scenario than its opposite, but more detailed natural history and ecological information will be required to rigorously test the role of habitat shifts in the diversification of *Hogna* in Madeira, as well as to determine instances of parallel evolution in habitat and functional traits, as has been reported in *Hogna* in the Galapagos Is. (De Busschere et al. 2010; De Busschere et al. 2012).

### ﻿*Hognainsularum* and *H.maderiana*: one or two species?

**Figure 28.**
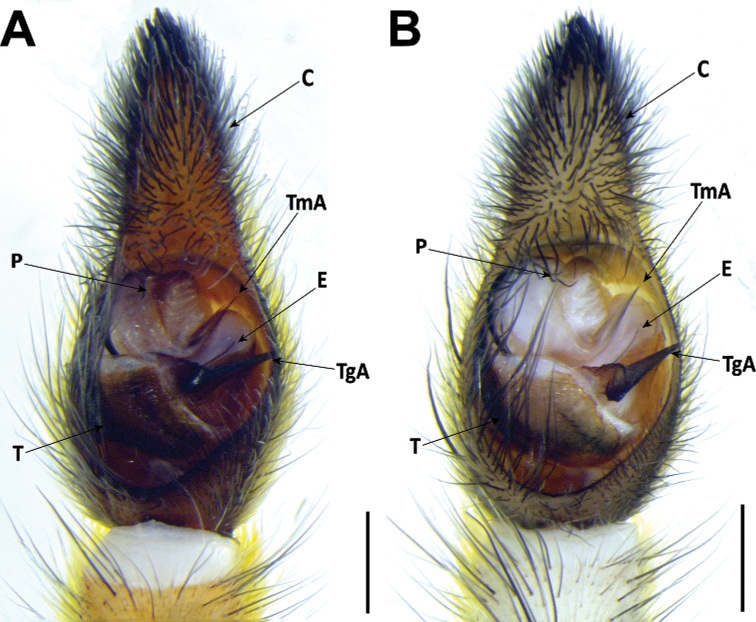
Unidentified male specimens belonging to the *H.maderiana* / *H.insularum* complex from Porto Santo. Left male pedipalps, ventral **A** CRBALC0328 **B** CRBALC0345. Scale bar: 0.5 mm (**A**).

**Figure 29.**
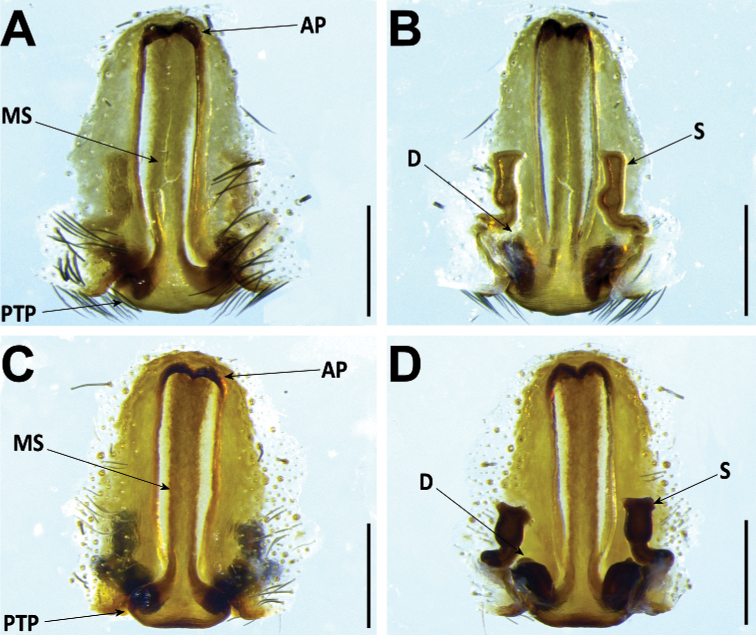
Unidentified female specimens belonging to the *H.maderiana* / *H.insularum* complex from Porto Santo. Female genitalia **A, B** CRBALC0329 **A** epigyne, ventral **B** vulva, dorsal **C, D** CRBALC0346 **C** epigyne, ventral **D** vulva, dorsal. Scale bar: 0.5 mm (**A**).

The species pair *H.insularum* and *H.maderiana* poses a taxonomic and evolutionary conundrum. Our molecular data were unable to establish boundaries between the large specimens of *Hogna* from the island of Porto Santo showing orange pilosity, identified using traditional diagnoses as *H.maderiana*, and the smaller specimens, without such pilosity, identified as *H.insularum*. Re-examination of morphological data suggested the existence of a continuum of phenotypic traits between the two extremes represented by specimens univocally referred to as either *H.maderiana* or *H.insularum*. Several specimens of intermediate size in Porto Santo (Figs 28, 29) showed clear yellowish to orange pilosity in anterior legs (colour fades to yellow after depositing specimen in ethanol), but not as dense as in the larger specimens. Furthermore, we were able to spot the usual dark reticulate pattern on the legs of these specimens, unlike in the large specimens, which are dark, bearing no traces of reticulated patterns (Fig. 27). We considered these specimens tentatively as “unidentified” (sp.). At the other extreme, the smaller specimens from Porto Santo, putatively identified as *H.insularum*, lacked orange setae, but showed yellowish to whitish setae. Certainly, although a remarkable size difference stands between the smallest specimens identified as *H.insularum* and the largest specimens identified as *H.maderiana*, similar wide intraspecific variation in size has been observed in other *Hogna* species, for example the Mediterranean species *H.radiata* (Latreille, 1817), which may range in size from 10 to 25 mm (Moya-Laraño, pers. comm.). Regarding male genitalic characters, Wunderlich (1992) proposed that the presence of a concavity in the tegulum as a diagnostic trait for *H.maderiana*. This trait is readily apparent in the large specimen we photographed (Fig. 21A, white arrow), but not in the unidentified specimens of intermediate size (Fig. 28). This feature, however, could be the result of a mechanical constraint associated to the role of the tegulum in supporting the tegular apophysis in large specimens. Similarly, although the embolus is usually smoothly curved in both *H.maderiana* and *H.insularum*, the actual degree of curvature may also vary across specimens (e.g., specimen CRBALC0328 bears a straighter embolus compared to other specimens, Fig. 28A). On the other hand, the SEM imaging revealed the presence in the embolic area of *H.insularum* (specimen CRBALC0310, from Porto Santo, Fig. 15E, F) of the loose membranous subterminal apophysis, indistinct under the microscope, which is not present in *H.maderiana* (Fig. 21D). However, caution should be taken as this might be an artifact of suboptimal drying process of the former specimen, which could have detached the pars pendula from the apposition with the embolus. Also, by looking at Fig. 15F, we can see that the subterminal apophysis is folded in a way that could plausibly accompany the embolus over a larger length. A similar pattern of intermediate forms can also be recognised among female specimens. Although *H.maderiana* specimens may be diagnosed by long median septum of the epigyne, the longest among Madeiran *Hogna* (Fig. 21E, F), a significant correlation exists between epigyne size (length/width at base) and body size (Pearson’s R = 0.71, p < 0.05, from a sample of 12 females), as revealed by the unidentified specimens from Porto Santo and females identified as *H.insularum*. Regardless of the actual length, the overall shape of the lateral borders of the anterior pockets is very similar across both taxa, showing parallel borders. Interestingly, the single adult *H.insularum* female available from Madeira, a population with distinct and exclusive mtDNA haplotypes, showed a slightly different epigynal shape (Fig. 16C). A similar relationship with body size is also observed in the shape of the spermathecae, which are pear-shaped in larger specimens (Fig. 21F), but from ovoid, to pear-shaped and rounded in smaller *H.insularum* specimens (Fig. 16B, D, F, H). Finally, regarding habitats, the largest specimens identified as *H.maderiana* are usually found in open, grassy meadows, while smaller specimens identified as *H.insularum* can be found both in the former habitat but also in shady (secondary) forest.

With the data at hand, it may seem advisable to merge both names into the same species. However, by doing so we might be concealing some interesting biological processes. For instance, hybridisation among close relatives have been uncovered between closely related *Hogna* species from the Galapagos islands (De Busschere et al. 2015). Hypothetically, introgression of adaptive genes among populations on different Galapagos islands may have contributed to the parallel evolution of similar ecological preferences. The ability of *Hogna* endemic species in Madeira to disperse between islands, which could promote introgression, is evident by the surprising finding of immature specimens originally identified as *H.insularum*, but that both mitochondrial and nuclear DNA suggested they belong to *H.ingens*, supposedly endemic to Desertas. Similar conflicting signals between different sources of evidence, namely morphology and molecules, may also arise in recently diverged species or species with large ancestral population sizes, as exemplified by wolf spiders in the genus *Pardosa* (Ivanov et al. 2021). Discerning alternative scenarios will require the future integration of large-scale population sampling with novel genome wide screening (e.g., ddRADSeq) methods.

### ﻿Conservation status

As for other taxa in the archipelago (Crespo et al. 2014, 2021; Cardoso et al. 2018e), the combination of restricted range and degrading habitat has led several species of endemic *Hogna* to be considered as threatened. While many seem to be relatively widely distributed and abundant, three species are of concern.

*Hognaingens*, the Desertas wolf spider, is limited to a single valley in the northern tip of Deserta Grande and was recently subjected to a reduction of 80% of its range in a few years (Crespo et al. 2014b), leading to a classification of Critically Endangered. A habitat recovery program is underway and several ex-situ populations are now guaranteeing its future survival. Recent data suggest that the habitat recovery is resulting in the recovery of the spider population to previously affected areas. If this is confirmed the status might improve and the status should be revised in the near future.

*Hognanonannulata* seems to be restricted to a small range in the south coast of the island of Madeira. With increasing urban pressure, it is possible that the status of Critically Endangered is warranted for the species. More information should be collected however, as contrary to most other regions in the archipelago, the area was never subject to extensive sampling.

*Hognaisambertoi* sp. nov. is the third species of conservation concern, given its small range and possible threat from aridification of the two locations from where it is known. The scarce available data of its life cycle, with adults emerging during November and December, warrant a monitoring program to confirm a possible status of Endangered.

We strongly recommend the rapid collection of data that can confirm or not the status of *H.nonannulata* and *H.isambertoi*, by focusing on monitoring programs of the southern coast of the Island of Madeira and overwintering in the southern tip of Deserta Grande and Bugio. If confirmed, these species would benefit from both habitat recovery programs and ex-situ conservation as is proving successful for *H.ingens*.

## ﻿Conclusions

Our study underlines the importance of the integration of different lines of evidence to fully understand the origin and diversification of species endemic to oceanic islands. Madeiran *Hogna* colonised the archipelago at a time of global expansion of grasslands and subsequently diversified throughout the archipelago into a variety of forms and sizes. Yet, the boundaries of some species are ill-defined and there are cases where both morphological and molecular suggest complex underlying evolutionary processes.

We tackled nomenclatural issues by revising old types and descriptions, describing a new species, and providing the first molecular data for Madeiran *Hogna*. The newly collected data confirmed the localised distribution and narrow range of some species. Our study sets the stage for the urgent implementation of conservation measures for the protection of these remarkable endemic species.

## Supplementary Material

XML Treatment for Hogna

XML Treatment for Hogna blackwalli

XML Treatment for Hogna ferox

XML Treatment for Hogna heeri

XML Treatment for Hogna ingens

XML Treatment for Hogna insularum

XML Treatment for Hogna isambertoi

XML Treatment for Hogna maderiana

XML Treatment for Hogna nonannulata

## Supplementary materials

Supplementary material 1 Table S1. Primers used for amplification Data type docx. file Explanation note Primers used for amplification. https://binary.pensoft.net/file/647010This dataset is made available under the Open Database License (http://opendatacommons.org/licenses/odbl/1.0/). The Open Database License (ODbL) is a license agreement intended to allow users to freely share, modify, and use this Dataset while maintaining this same freedom for others, provided that the original source and author(s) are credited.Luís C. Crespo, Isamberto Silva, Alba Enguídanos, Pedro Cardoso, Miquel Arnedo

Supplementary material 2 Tables S2, S3 Data type Materials Explanation note Spreadsheet containing all the studied specimens, their collection data and the checklist of amplified genes, as well as the outgroup taxa accession numbers. https://binary.pensoft.net/file/647011This dataset is made available under the Open Database License (http://opendatacommons.org/licenses/odbl/1.0/). The Open Database License (ODbL) is a license agreement intended to allow users to freely share, modify, and use this Dataset while maintaining this same freedom for others, provided that the original source and author(s) are credited.Miquel Arnedo

Supplementary material 3 Figure S1 Data type Phylogenetic (tiff. image) Explanation note Full best Maximum Likelihood tree of Lycosoidea, inferred with IQTREE2 after selecting the best partition scheme and evolutionary models. Nodes are split in three sections, representing the different methods. Support on nodes should be read as follows: black: ML ultrafast boostrap and BI posterior probability ≥ 0.95, MP Jackknife ≥ 0.7; grey: ML Ultrafast Bootstrap and BI posterior probability < 0.95, MP Jackknife < 0.7; white: unrecovered node. https://binary.pensoft.net/file/647012This dataset is made available under the Open Database License (http://opendatacommons.org/licenses/odbl/1.0/). The Open Database License (ODbL) is a license agreement intended to allow users to freely share, modify, and use this Dataset while maintaining this same freedom for others, provided that the original source and author(s) are credited.Miquel Arnedo, Luís C. Crespo
