# Supplementary material for: ﻿Island hoppers: Integrative taxonomic revision of Hogna wolf spiders (Araneae, Lycosidae) endemic to the Madeira islands with description of a new species
Source: Zookeys. 2022 Feb 16;1086:84–135. doi: 10.3897/zookeys.1086.68015 (PMC8866340; doi:10.3897/zookeys.1086.68015)
Supplement: Supplementary material 1 — Table S1. Primers used for amplification [file zookeys-1086-084-s001.docx]

Supplementary file 1A

Table S1. Primers used for amplification.

| Locus | Primer name | Primer Sequence | Reference |
| --- | --- | --- | --- |
| COI | C1-J-1490 | GGTCAACAAATCATAAAGATATTGG | (Folmer et al. 1994) |
|  | C1-N-2198 | TAAACTTCAGGGTGACCAAAAAATCA | (Folmer et al. 1994) |
|  | C1-N-2191 | CCCGGTAAAATTAAAATATAAACTTC | (Simon et al. 1994) |
|  | C1-J-1751 | GGATCACCTGATATAGCATTCCC | (Simon et al. 1994) |
| 16S | LR-N-13398 | CGCCTGTTTATCAAAAACAT | (Simon et al. 1994) |
|  | LR-J-12864 | CTCCGGTTTGAACTCAGATCA | (Palumbi 1996) |
| NAD1 | LR-N-12945 | CGACCTCGATGTTGAATTAA | (Hedin 1997) |
|  | N1-J-12373 | CTTCGTATAGATCCTARTTGDCTRTATT | (Macías-Hernández, Oromí & Arnedo, 2008) |
|  | N1-J-12261 | TCRTAAGAAATTATTTGAGC | (Hedin 1997) |
| 28S | 28SO | GACCCGTCTTGAAACACGGA | (Hedin and Maddison 2001) |
|  | 28SB | TCGGAAGGAACGAGCTAC | (Whiting et al. 1997) |
|  | 28SC | GGTTCGATTAGTCTTTCGCC | (Hedin and Maddison 2001) |
| H3 | H3F | ATGGCTCGTACCAAGCAGACVGC | (Colgan et al. 1998) |
|  | H3R | ATATCCTTRGGCATRATRGTGAC | (Colgan et al. 1998) |
| ITS-2 | ITS-5.8S | GGGACGATGAAGAACGGAGC | (White et al. 1990) |
|  | ITS-28S | TCCTCCGCTTATTGATATGC | (White et al. 1990) |
| 12S | 12SR-J-14199 | TACTATGTTACGACTTAT | (Kambhampati and Smith 1995) |
|  | 12SR-N-14594 | AAACTAGGATTAGATACCC | (Kambhampati and Smith 1995) |

Supplementary file 1B: PCR amplification settings for all studied loci. The number of cycles used was 35 for all loci.

|  | COI | 16S, NAD1 | 28S | H3 | 12S | ITS-2 |
| --- | --- | --- | --- | --- | --- | --- |
| Denaturation | 94 °C, 5 m | 94 °C, 5 m | 94° C, 5 m | 94° C, 5 m | 94° C, 5 m | 94° C, 5 m |
| Cycles for annealing and initial extension | 94 °C, 30 s | 94 °C, 30 s | 94 °C, 30 s | 94 °C, 30 s | 94 °C, 30 s | 94 °C, 30 s |
|  | 42 °C, 35 s | 45 °C, 35 s | 48 °C, 35 s | 45 °C, 35 s | 42–45 °C, 35 s | 50 °C, 35 s |
|  | 72 °C, 45 s | 72 °C, 45 s | 72 °C, 1 m | 72 °C, 45 s | 72 °C, 45 s | 72 °C, 45 s |
| Final extension | 72 °C, 5 m | 72 °C, 5 m | 72 °C, 10 m | 72 °C, 5 m | 72 °C, 5 m | 72 °C, 5 m |
