# Supplementary figures and images for: ﻿Island hoppers: Integrative taxonomic revision of Hogna wolf spiders (Araneae, Lycosidae) endemic to the Madeira islands with description of a new species
Source: Zookeys. 2022 Feb 16;1086:84–135. doi: 10.3897/zookeys.1086.68015 (PMC8866340; doi:10.3897/zookeys.1086.68015)

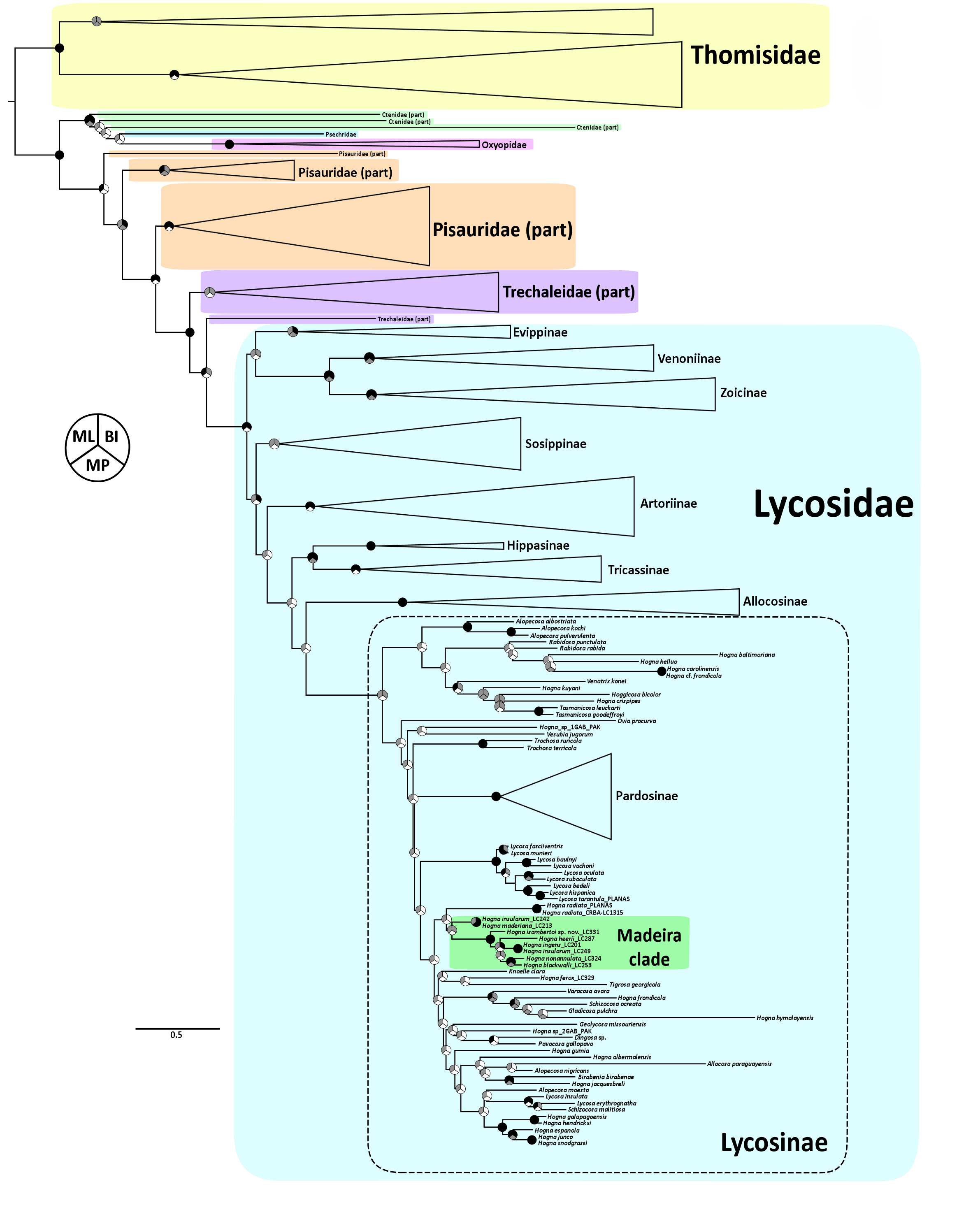

Supplement: Supplementary material 3 — Figure S1 [file zookeys-1086-084-s003.tif]
